# PhosContext2vec: a distributed representation of residue-level sequence contexts and its application to general and kinase-specific phosphorylation site prediction

**DOI:** 10.1038/s41598-018-26392-7

**Published:** 2018-05-29

**Authors:** Ying Xu, Jiangning Song, Campbell Wilson, James C. Whisstock

**Affiliations:** 1 Faculty of Information Technology, Monash University, Melbourne, VIC 3800 Australia; 2 Monash Centre for Data Science, Faculty of Information Technology, Monash University, Melbourne, VIC 3800 Australia; 3 Infection and Immunity Program, Biomedicine Discovery Institute and Department of Biochemistry and Molecular Biology, Monash University, Melbourne, VIC 3800 Australia; 4 ARC Centre of Excellence in Advanced Molecular Imaging, Monash University, Melbourne, VIC 3800 Australia

## Abstract

Phosphorylation is the most important type of protein post-translational modification. Accordingly, reliable identification of kinase-mediated phosphorylation has important implications for functional annotation of phosphorylated substrates and characterization of cellular signalling pathways. The local sequence context surrounding potential phosphorylation sites is considered to harbour the most relevant information for phosphorylation site prediction models. However, currently there is a lack of condensed vector representation for this important contextual information, despite the presence of varying residue-level features that can be constructed from sequence homology profiles, structural information, and physicochemical properties. To address this issue, we present PhosContext2vec which is a distributed representation of residue-level sequence contexts for potential phosphorylation sites and demonstrate its application in both general and kinase-specific phosphorylation site predictions. Benchmarking experiments indicate that PhosContext2vec could achieve promising predictive performance compared with several other existing methods for phosphorylation site prediction. We envisage that PhosContext2vec, as a new sequence context representation, can be used in combination with other informative residue-level features to improve the classification performance in a number of related bioinformatics tasks that require appropriate residue-level feature vector representation and extraction. The web server of PhosContext2vec is publicly available at http://phoscontext2vec.erc.monash.edu/.

## Introduction

Phosphorylation is the most common type of protein post-translational modification (PTM), which plays an important role in almost all cellular processes in eukaryotes^1,2^. Accurate prediction of kinase-mediated phosphorylation sites is important for functional annotations of target substrates and elucidation of cellular signaling pathways underlying such phosphorylation events. A widely accepted concept during the development of computational methods for phosphorylation site prediction is that most substrate proteins can be phosphorylated at specific sites with sequential and structural motifs or patterns^3,4^. Since sequence information is more accessible than structural information, high-throughput and accurate phosphorylation site prediction tools based on sequence information are highly desirable. According to previous studies, the prediction of phosphorylation sites is addressed in two ways, i.e. general phosphorylation site prediction and kinase-specific phosphorylation site prediction. The former only considers the difference between different phosphorylation site types, for example, serine, threonine and tyrosine phosphorylation sites^5,6^, while the latter distinguishes different kinase-regulated phosphorylation sites in a kinase-specific manner^3,5,7–10^.

Word embedding is a useful technique in natural language processing that maps words to numeric representations in the vector space^11^. One of the most successful word embedding-based models is the *word2vec* model for generating distributed representations of words and phrases^12^. It was proposed to automatically generate condensed feature vectors for words and phrases by using a generative model that is pre-trained based on large-scale sequence databases. Asgari and Mofrad further proposed a continuous distributed representation for biological sequences, termed *ProtVec*, by summing up the distributed representations of biological words in each biological sequence^13^. It was used as the input feature to address sequence- or segment-level prediction tasks such as annotating each protein sequence/segment as ordered/disordered or assigning each protein sequence to a protein family. Inspired by these representation techniques, in this study, we propose a distributed representation of residue-level sequence contexts, termed *context2vec*, for addressing residue-level prediction tasks, such as predicting the status of each residue as being phosphorylated or non-phosphorylated. More specifically, to take into consideration the dependence between phosphorylation sites and their sequential contextual patterns, we generated the distributed representation of the local sequence context for each potential phosphorylation site and then used it as a contextual feature vector to predict both general and kinase-specific phosphorylation sites. Different from the *ProtVec* method which maps a whole biological sequence to its distributed representation at the sequence-level, *context2vec* addresses an important issue related to the representation of residue-level sequence contexts and the selection of an appropriate task-dependent contextual window size. In particular, *context2vec* enables the representation of more enriched contextual information than sequence-level features (such as *ProtVec*)^13^ and provides contextual information complementary to other commonly used residue-level features, such as position-specific scoring matrixes and secondary structures^14–16^.

Specifically, we propose two different implementation strategies to generate distributed contextual feature vectors for phosphorylation site prediction and compared their predictive performance. Firstly, we apply the *word2vec*-based summation strategy of *ProtVet* to residue-level sequence contexts, referred to as *context2vec*^*add*^ in this paper. Secondly, we apply the *doc2vec* model^17^ to infer the distributed representation of residue-level sequence contexts directly from a pre-trained distributed memory network, referred to as *context2vec*^*inference*^. The major difference between *context2vec*^*add*^ and *context2vec*^*inference*^ is that the latter considers the order of amino acid sequences in the context of each residue, while the strategy of representation summation used by the former implementation does not consider this important aspect. Nevertheless, a disadvantage of *context2vec*^*inference*^ is that the quality of the inferred representations may decrease slightly during the process of approximate inference. In this study, we applied both *context2vec*^*add*^ and context2vec^*inference*^ to generate the prediction models of phosphorylation sites (termed as PhosContext2vec), benchmarked the performance of PhosContext2vec for both general and kinase-specific phosphorylation site predictions, and constructed the corresponding best-performing models for each type of phosphorylation sites and kinase families.

To facilitate community-wide applications, we made available an online web server of PhosContext2vec for generating distributed representation of residue-level contextual feature vectors and predicting general and kinase-specific phosphorylation sites. To the best of our knowledge, this is the first time that the distributed representation of protein sequences is applied to address the problem of protein PTM (phosphorylation in this study) site prediction.

## Methods

### Datasets

In order to assess the performance of PhosContext2vec in comparison with other existing methods, we conducted cross-validation and independent tests for both general and kinase-specific phosphorylation site predictions.

For general phosphorylation site prediction, we used the same training and independent test datasets of annotated Serine (S), Threonine (T) and Tyrosine (Y) phosphorylation sites that were originally introduced in PhosphoSVM^6^. The training dataset, termed PELM, was constructed from the database Phospho.ELM (Version 9.0)^18^ which included experimentally verified phosphorylation sites in animals such as *Homo sapiens*, *Mus musculus*, *Drosophila melanogaster*, and *Caenorhabditis elegans*. It had 6632, 3226, and 1,392 proteins containing 20,960, 5,684, and 2,163 phosphorylation sites for S, T, and Y sites, respectively. The independent test set, named PPA, was extracted from the database PhosphAt (Version 3.0)^19^ which only contained plant phosphorylation sites from *Arabidopsis thaliana*. The PPA dataset contained 3,037, 1,359 and 617 substrate proteins with 5,449, 1,686, and 676 annotated phosphorylation sites, for S, T, and Y sites, respectively.

For kinase-specific phosphorylation site prediction, we combined phosphorylation site data obtained from Phospho.ELM^18^ and UniProt^20^ and constructed training and independent test datasets according to the following steps. Firstly, we downloaded 555,594 reviewed proteins from the UniProt database and extracted all the proteins that had at least one annotated phosphorylation site, resulting in 14,458 proteins in total. Next, we collected triple-record annotations (i.e. containing *protein*, *phosphorylation site position*, and *kinase* annotations) from UniProt for the 14,458 proteins and removed 2,155 triple-record annotations that were labeled as ‘by similarity’ in UniProt. The resulting 56,772 triple-record annotations contained 43,785 phosphorylated S sites, 10,397 phosphorylated T sites, and 4,711 phosphorylated Y sites, among which 7,021, 2,515, and 2,066 were respectively annotated with kinase types. Similarly, we extracted the triple-record annotations from the Phospho.ELM (version 9.0) database, resulting in 43,027 phosphorylated S sites, 9,556 phosphorylated T sites, and 4,723 phosphorylated Y sites, among which 2,961, 943, and 1,031 sites were respectively annotated with the corresponding kinase types.

In order to combine triple-record annotations from UniProt and Phospho.ELM, we employed the following procedures: (a) we cross-checked and renamed proteins extracted from the UniProt database whose sequences have been updated compared to those in the Phospho.ELM database; (b) we manually corrected the kinase names according to the hierarchical structure of the kinase groups, families, subfamilies, and types (Refer to Table S1 in the GPS 2.0 paper)^9^; (c) we removed redundant entries that were included in both two databases, and d) we excluded kinases that had less than 20 triple-record annotations. In total, we obtained consolidated phosphorylation sites for 138 kinase groups, families, subfamilies and protein kinases. In this study, we only performed cross-validation and independent tests on five kinase families that had more than 500 triple-record annotations. These included AGC/PKC, AGC/PKA, CMGC/CK2 (previsouly classified as member of the kinase group Other), CMGC/CDK, and TK/SRC with 962, 897, 668, 628, and 631 triple-record annotations, respectively. To obtain the training and testing datasets, we divided the triple-record annotations into five subsets, among which four were used as the positive samples in training data and the remaining one was used as the positive samples in testing data. At the same time, S, T, and Y sites that were not annotated as phosphosites were treated as negative samples. A statistical summary of the finally curated training and independent test datasets is shown in Tables S1 and S2 in the Supplementary Material.

The curated phosphorylation site datasets are highly imbalanced, where the negative samples were hundreds of times more than the positive samples. In previous studies, the issue of imbalanced data set was addressed by down-sampling the negative samples^5–7,21^ or augmenting the positive samples^22^. For down-sampling, the negative samples can be randomly sampled from all [S, T and Y] sites that were not annotated as phosphorylation sites^6,7,21^. They can also be sampled from non-phosphorylated S, T, or Y sites depending on the type of site or kinase the model was trained for^5^. In this study, for the purpose of training, we included all [S, T, and Y] sites, that were not annotated as phosphosites, as negative samples for performing the down-sampling. Negative samples were randomly selected to train the prediction models with a ratio of 1:1 between the positives and negatives. For the purpose of independent test, we included S, T or Y sites depending on the type of sites or the kinase the model was trained for, without down-sampling. For example, for the model that was trained to predict phosphorylated Y sites, we only included all Y sites, which were not annotated as phosphosites, as negative samples. While for the model that was trained to predict sites that are specifically phosphorylated by the AGC/PKA kinase, we included non-phosphorylated S and T sites as negative samples.

### Cross-validation and independent tests

We performed 10-fold cross validation tests using each of the aforementioned benchmark datasets where, in each iteration, nine folds were used for training the model while the remaining fold was used to validate the prediction performance of the trained model. This process was repeated 10 times so that each fold was used for both model training and validation. Based on 10-fold cross-validation results, we selected the model that achieved the best performance on the validation set and further tested its performance on the independent test datasets. Different from the cross-validation for which down-sampling was used to ensure selection of equal numbers of positive and negative samples, independent tests were performed against imbalanced datasets which contained more negative samples than positive samples. Therefore, although the performance on independent tests appeared to be worse than that in cross-validation tests, it resembled a real-world scenario more closely.

### Performance evaluation

We evaluated the prediction performance of constructed models using four measurements, including sensitivity (Eq. 1), specificity (Eq. 2), the Matthews coefficients of correlation (MCC) (Eq. 3), and the area under the ROC curve (AUC)^9^. Except for the AUC, all the other three measures rely on the selection of the prediction cut-off thresholds for models to generate the final classification outcome. Because different predictors produced different ranges of predicted probability scores, we used the False Positive Rate (PFR) to determine positive predictions. According to GPS 3.0^3^, the low, medium and high cut-off FPRs for S and T sites were respectively set as 2%, 6%, and 10%, while the low, medium and high cut-off FPRs for Y sites were set as 4%, 9%, and 15%, respectively.

1 $${\rm{Sensitivity}}=\frac{TP}{TP+FN}$$

2 $${\rm{Specificity}}=\frac{TN}{TN+FP}$$

3 $${\rm{MCC}}=\frac{TP\times TN+FP\times FN}{\sqrt{(TP+FP)(TP+FN)(TN+FP)(TN+FN)}}$$

where *TP*, *TN*, *FP* and *FN* represent the numbers of true positives, true negatives, false positives, and false negatives, respectively.

### Distributed representation of protein sequences and segments

We used a five-step procedure to generate the distributed representation of protein sequences and segments. Our procedure is detailed as follows:

1. We constructed the training dataset from a large protein sequence database in order to train the model to generate distributed representation of protein sequences. A total number of 551,704 reviewed protein sequences were downloaded and extracted from the UniProt database^20^ in Aug, 2016;
2. For each protein sequence in the dataset, we performed the overlapping *n*-gram split which produced a list of *n*-gram biological words^13^. Asgari and Mofrad showed that 3-gram split led to the best performance^13^; we thus produced all the 3-gram biological words for all the extracted 551,704 protein sequences based on the 3*-*gram split;
3. We fed the 3-gram biological words to the *doc2vec* algorithm to train a vector-generating model consisting of three components, including a trained distributed memory network N for future inference, a database *DB*_*w*_ that mapped the biological words to their pre-trained vectors, and a database *DB*_*p*_ that mapped protein sequences to their pre-trained vectors. The *doc2vec* algorithm in Gensim^12^ was used in this training process;
4. For any target protein sequence or sequence segment, we split it into *n*-gram biological words in the same manner as described in step (2);
5. The distributed representation of the target protein sequence or sequence segment was then generated using two implementation strategies: (i) The representations of all biological words in the target protein sequence were directly fetched from the database *DB*_*w*_ and summed up to form the protein sequence representation, termed as *prot2vec*^*add*^, and (ii) The resulting list of biological words was fed to the pre-trained model N to generate the other type of protein sequence representation, termed as *prot2vec*^*inference*^.

At the step (2), protein sequences can be split into *overlapping* or *non-overlapping n*-gram biological words^13^. We tested these two splitting strategies and found that a better performance was achieved when splitting the sequences into *overlapping* biological words. At the step (3), the hyper-parameters for the *doc2vec* algorithm were set as follows. The hidden unit size was set as 100, the window size set as 25, the initial training learning rate set as 0.25, the parameter for the negative sampling set as 5, and the iteration number set as 400. At the final step, the *prot2vec*^*add*^ representation was effectively equivalent to *ProtVec* that was originally introduced by Asgari and Mofrad^13^. To facilitate understanding and comparison between different methods, we renamed *ProtVec* to *prot2vec*^*add*^ (‘add’ indicates addition or summation), so that it could be better compared with and distinguished from *prot2vec*^*inference*^ proposed in this study, from the perspective of feature vector generation strategies.

### Feature vector construction for phosphorylation site prediction models

We constructed prediction models for both general and kinase-specific phosphorylation sites using the Support Vector Machine (SVM) algorithm^23^. For each of the three phosphorylation site types S, T, and Y, and each of the five kinase families AGC/PKC, AGC/PKA, CMGC/CK2, CMGC/CDK, and TK/SRC, an independent SVM model was constructed based on the proposed contextual feature vector in conjunction with six other residue-level feature groups. The six residue-level features were the Shannon entropy, the relative entropy, protein disordered property, secondary structures, Taylor’s overlapping properties, and the average cumulative hydrophobicity, which are briefly described below:

1. The Shannon entropy. It is a feature used for quantifying the conservation of potential phosphorylation sites^24^. It is calculated based on the weighted observed percentage (WOP), which can be generated by PSI-BLAST^16^;
2. The relative entropy. It measures the conservation of amino acids compared to the background distributions, such as BLOSUM62^25^. It is also calculated based on the WOP;
3. Protein disorder information (DISO). Certain regions of the protein do not form stable structures, and are called disordered regions. Previous studies indicate that incorporating protein disorder information is often useful for improving the prediction performance^10,26–30^. In this study, the protein disorder information was predicted using the DISOPRED3 program^15^;
4. Protein secondary structure (PSS). It includes helix, strands and coil assignments in proteins. We used the PSIPRED program^31^ to predict the secondary structure information for each protein sequence;
5. The Taylor’s overlapping property (OP). It describes amino acid groups with respect to physiochemical properties. Each amino acid is encoded into 10 bits representing 10 physiochemical properties respectively^32^;
6. The average cumulative hydrophobicity (ACH). A varying sliding window of sizes 3, 5, 7, …, 21 was used to extract the local sequence environment surrounding a potential phosphorylation site. The average hydrophobicity of all amino acids in the local window was calculated^33^. In this study, the Sweet and Eisenberg hydrophobicity index^34^ was used to encode this feature group.

The generation of contextual feature vector was based on the aforementioned distributed representation of protein sequences and segments. More specifically, we generated the distributed representation of residue-level sequence contexts within specific contextual window sizes. This residue-level sequence context was referred to as *PSP* (*m*, *n*) in previous studies, namely the Phosphorylation Site Peptide, which was composed of *m* upstream residues and *n* downstream residues of the target site^21^. In this study, we only considered the situation where an equal number of residues from the upstream and downstream of the target site was used, i.e. *m* = *n*. Thus, the contextual feature vector for a potential phosphorylation site *r*_*i*_ can be generated as follows:

1. We extracted the contextual window of size *w*_*i*_ = 2*m* + 1 for a residue *r*_*i*_, which consisted of *m* upstream residues and *n* = *m* downstream residues. The resulting contextual window was denoted as *c*_*i*_ = *r*_*i*−*m*+1_, *r*_*i*−*m*+1_, …, *r*_*i*_, …, *r*_*i*+*m*−1_, *r*_*i*+*m*_;
2. We performed *n*-gram split of the contextual window *w*_*i*_, resulting in a list of biological words;
3. We generated the distributed representation of *w*_*i*_ in two different ways: (i) by summing up the distributed representation of all its biological words, named *context2vec*^*add*^, and (ii) by feeding the list of biological words to the pre-trained distributed memory network N, named *context2vec*^*inference*^.

**Figure 1:**
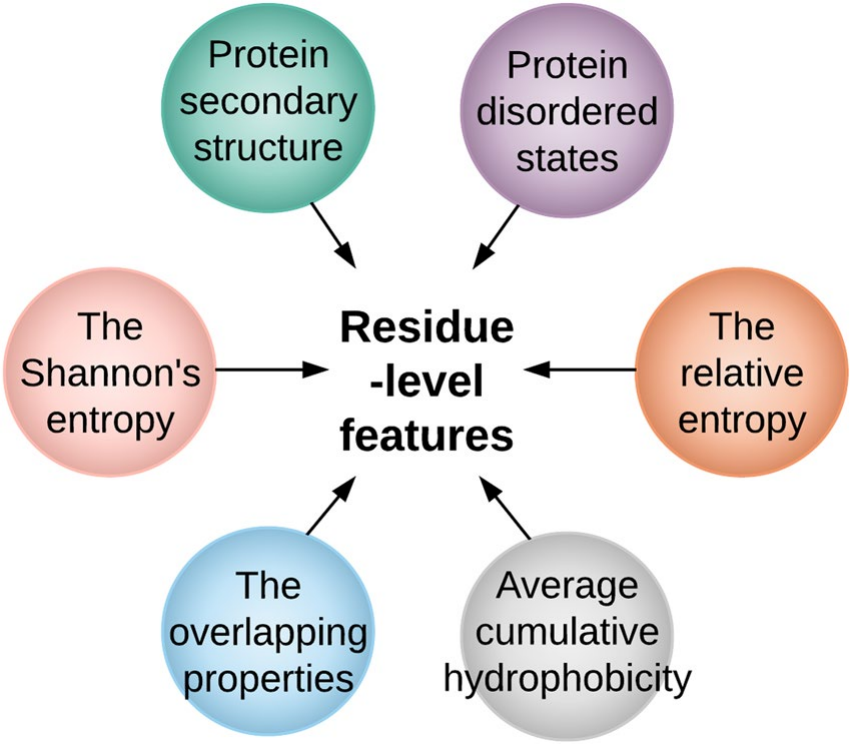
Extraction of residue-level feature groups for phosphorylation site prediction.

**Figure 2:**
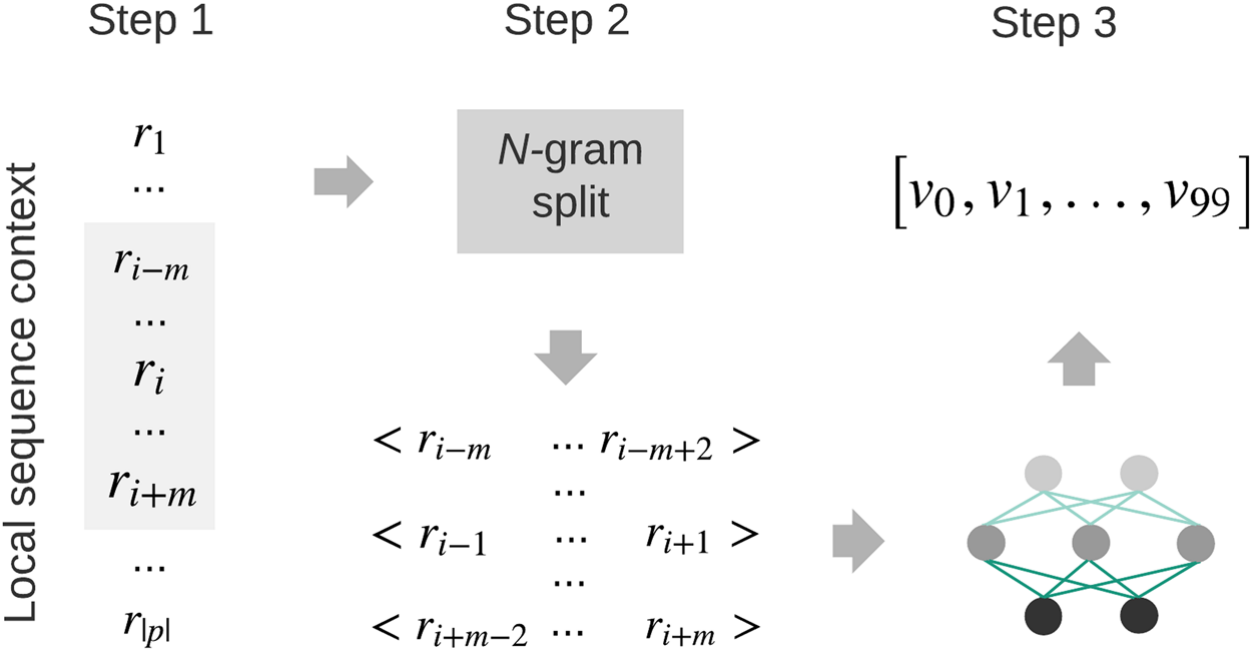
Generation of the distributed representation of residue-level sequence contexts for phosphorylation site prediction.

The final feature vector of the potential phosphorylation site *r*_*i*_ was constructed by stacking all the seven aforementioned groups of feature vectors together, resulting in a 126-dimensional feature vector. Figure 1 demonstrates the six groups of residue-level features and Fig. 2 provides a flowchart illustrating how the residue-level sequence contexts are represented as *context2vecs* for phosphorylation site prediction.

## Results

We conducted comprehensive benchmarking experiments to compare the performance of PhosContext2vec and seven existing predictors for phosphorylation site prediction. More specifically, in the case of general phosphorylation site prediction, the prediction performance of PhosContext2vec was compared to that of GPS 3.0^3,9,21^, MusiteDeep^35^, Musite 1.0^5^, and NetPhos 3.1^22,36^. While in the case of kinase-specific phosphorylation site prediction, the performance of PhosContext2vec was compared to that of GPS 3.0, MusiteDeep, Musite 1.0, NetPhos 3.1, KinasePhos 2.0^7,8^, PhosphoPredict^10^, and PhosphoPick^37^. The corresponding algorithms, training data sources and features used by these methods are summarized in Table 1.

**Table 1:** Summary of the algorithms, training data sources and feature groups used by different phosphorylation site predictors on the independent test.

Predictors	Algorithms	Data sources	Feature groups	G/K*
GPS 3.0	Hierarchical clustering^9^	Phospho.ELM^18^, PhosphoBase^49^	Phosphorylation site peptide (PSP) sequence similarities^21^	G/K
MusiteDeep	Deep CNN, LSTM^50^	UniProt/Swiss-prot^20^, RegPhos^51^	One-of-K coding for 16 upstream and downstream residues	G/K
Musite 1.0	Ensemble learning^52^	Phospho.ELM, UniProt, PhosphoPep^53^, PhosphAt^19^	K-nearest neighbour (KNN) scores^5^, disorder states, and amino acid frequencies^54^	G/K
NetPhos 3.1	Neural networks	PhosphoBase	Convolutional sparse encoding^55^ of local sequence contexts	G/K
KinasePhos 2.0	SVM^23^	PhosphoBase, UniProt^20^	Local sequence patterns and local coupling patterns^8^	K
PhosphoPredict	Random forest	Phospho.ELM	Amino acid type, PSS, DISO, solvent accessibility, and various protein functional features^10^	K
PhosphoPick	Bayesian networks^56^	Phospho.ELM, HPRD^57^	Protein-protein interactions^58^ and protein cell-cycle types^59^	K
PhosContext2vec	SVM	Phospho.ELM, UniProt	The Shannon entropy, the relative entropy, PSS, DISO, OP, ACH, and distributed contextual feature vectors	G/K

*G/K indicate *G*eneral and *K*inase-specific phosphorylation site prediction, respectively.

### The effect of incorporating the distributed representation of residue-level sequence contexts on the predictive performance

To characterize the effect of distributed representation of sequence contexts on both general and kinase-specific phosphorylation site prediction, we performed 10-fold cross-validation tests and evaluated the performance of models trained using residue-level features only (*Residue-level*), the *context2vec* generated with the inference strategy (*Context2vec*^*inference*^), the *context2vec* generated with the addition strategy (*Context2vec*^*add*^), the combination of residue-level features and *context2vec*^*inference*^ (*Residue-level* + *Context2vec*^*inference*^), the combination of residue-level features and *context2vec*^*add*^ (*Residue-level* + *Context2vec*^*add*^), and the combination of residue-level features and *ProtVec*^13^ (*Residue-level* + *prot2vec*^*add*^ (*ProtVec*)). Figures 1 and 2 show the generation of residue-level features and the *context2vec* representation, respectively. For the combined feature vectors, they were constructed by concatenating two feature vector representations for each target residue, respectively.

Tables 2 and 3 shows the performance results of different models trained using the aforementioned input feature vectors for general and kinase-specific phosphorylation site prediction, respectively. For *Context2vec*^*inference*^, *Context2vec*^*add*^, *Residue-level* + *Context2vec*^*inference*^, *and Residue-level* + *Context2vec*^*add*^ feature vectors, four different contextual window sizes (*ws*) 7, 11, 15, and 19 were used to extract the local sequence contexts based on which the *context2vec* features were generated. It should be noted that the *Residue-level* features were representative of the special case of contextual window size 0, while the *Residue-level* + *prot2vec*^*add*^ (*ProtVec*) represents the special case of infinite contextual window size (including the whole sequence). In Tables 2 and 3, the best predictive performance for each phosphorylation site type or kinase family achieved by *Context2vec-*only feature vectors (including *Context2vec*^*inference*^ and *Context2vec*^*add*^) and *Residue-level* + *Context2vec* feature vectors (including *Residue-level* + *Context2vec*^*inference*^ and *Residue-level* + *Context2vec*^*add*^) with respect to different contextual window sizes was highlighted by underline and bold, respectively.

**Table 2:** Performance comparison between models trained with and without distributed contextual feature vectors for general phosphorylation site prediction.

	*ws*	S	T	Y
*Residue-level*	*0*	0.842 +/− 0.008	0.896 +/− 0.007	0.933 +/− 0.007
*Context2vec* ^*inference*^	*7*	0.512 +/− 0.005	0.557 +/− 0.016	0.854 +/− 0.036
	*11*	0.845 +/− 0.005	0.861 +/− 0.011	0.871 +/− 0.016
	*15*	0.841 +/− 0.006	0.848 +/− 0.010	0.849 +/− 0.017
	*19*	0.835 +/− 0.006	0.836 +/− 0.014	0.833 +/− 0.017
*Context2vec* ^*add*^	*7*	0.852 +/− 0.008	0.884 +/− 0.009	0.909 +/− 0.011
	*11*	0.850 +/− 0.011	0.867 +/− 0.009	0.875 +/− 0.017
	*15*	0.843 +/− 0.013	0.853 +/− 0.011	0.856 +/− 0.018
	*19*	0.841 +/− 0.006	0.840 +/− 0.014	0.840 +/− 0.018
*Residue-level* + *Context2vec*^*inference*^	*7*	0.887 +/− 0.008	0.921 +/− 0.007	0.938 +/− 0.008
	*11*	0.889 +/− 0.008	0.926 +/− 0.008	0.938 +/− 0.008
	*15*	**0**.**889** +/− **0**.**008**	**0**.**927** +/− **0**.**009**	0.939 +/− 0.008
	*19*	0.887 +/− 0.008	0.926 +/− 0.008	**0**.**939** +/− **0**.**008**
*Residue-level* + *Context2vec*^*add*^	*7*	0.887 +/− 0.008	0.920 +/− 0.008	0.937 +/− 0.008
	*11*	0.892 +/− 0.008	0.927 +/− 0.007	0.938 +/− 0.008
	*15*	**0**.**892** +/− **0**.**008**	**0**.**929** +/− **0**.**006**	0.939 +/− 0.008
	*19*	0.891 +/− 0.008	0.929 +/− 0.005	**0**.**939** +/− **0**.**008**
*Residue-level* + *prot2vec*^*add*^ (*ProtVec*)	*inf*	0.842 +/− 0.010	0.901 +/− 0.006	0.938 +/− 0.007

The prediction performance was evaluated in terms of the average AUC score and the standard deviation.

**Table 3:** Performance comparison between models trained with and without distributed contextual feature vectors for kinase-specific phosphorylation site prediction.

	*ws*	AGC/PKA	AGC/PKC	CMGC/CDK	CMGC/CK2	TK/Src
*Residue-level*	*0*	0.915 +/− 0.013	0.898 +/− 0.022	0.799 +/− 0.040	0.839 +/− 0.040	0.956 +/− 0.011
*Context2vec* ^*inference*^	*7*	0.609 +/− 0.043	0.653 +/− 0.056	0.570 +/− 0.042	0.736 +/− 0.056	0.828 +/− 0.051
	*11*	0.918 +/− 0.013	0.862 +/− 0.031	0.890 +/− 0.029	0.885 +/− 0.048	0.913 +/− 0.014
	*15*	0.895 +/− 0.020	0.862 +/− 0.028	0.885 +/− 0.030	0.893 +/− 0.043	0.895 +/− 0.016
	*19*	0.880 +/− 0.018	0.865 +/− 0.026	0.872 +/− 0.031	0.889 +/− 0.044	0.875 +/− 0.022
*Context2vec* ^*add*^	*7*	0.885 +/− 0.025	0.809 +/− 0.022	0.903 +/− 0.030	0.814 +/− 0.055	0.931 +/− 0.010
	*11*	0.925 +/− 0.013	0.872 +/− 0.026	0.883 +/− 0.026	0.882 +/− 0.037	0.912 +/− 0.014
	*15*	0.906 +/− 0.015	0.882 +/− 0.023	0.872 +/− 0.034	0.898 +/− 0.032	0.893 +/− 0.017
	*19*	0.892 +/− 0.016	0.885 +/− 0.021	0.869 +/− 0.034	0.901 +/− 0.032	0.877 +/− 0.017
*Residue-level* + *Context2vec*^*inference*^	*7*	0.928 +/− 0.011	0.897 +/− 0.028	**0.925 +/− 0.018**	0.866 +/− 0.041	0.960 +/− 0.009
	*11*	**0.938 +/− 0.009**	0.909 +/− 0.027	0.908 +/− 0.018	0.907 +/− 0.037	0.964 +/− 0.008
	*15*	0.937 +/− 0.010	0.907 +/− 0.028	0.902 +/− 0.024	**0.919 +/− 0.034**	**0.966 +/− 0.007**
	*19*	0.937 +/− 0.011	**0.909 +/− 0.024**	0.899 +/− 0.025	0.917 +/− 0.033	0.965 +/− 0.008
*Residue-level* + *Context2vec*^*add*^	*7*	0.927 +/− 0.012	0.895 +/− 0.028	**0.907 +/− 0.020**	0.864 +/− 0.040	0.957 +/− 0.009
	*11*	0.939 +/− 0.010	0.911 +/− 0.023	0.896 +/− 0.020	0.911 +/− 0.034	0.962 +/− 0.009
	*15*	0.939 +/− 0.010	0.913 +/− 0.023	0.890 +/− 0.022	0.927 +/− 0.026	0.964 +/− 0.008
	*19*	**0.940 +/− 0.010**	**0.915 +/− 0.020**	0.894 +/− 0.024	**0.929 +/− 0.024**	**0.964 +/− 0.008**
*Residue-level* + *prot2vec*^*add*^(*ProtVec*)	*inf*	0.908 +/− 0.013	0.874 +/− 0.022	0.738 +/− 0.042	0.827 +/− 0.036	0.954 +/− 0.010

The prediction performance was evaluated in terms of the average AUC score and the standard deviation.

As clearly shown in Tables 2 and 3, the models trained using *Residue-level* + *Context2vec* features outperformed the models trained with *Residue-level* features across all three types of phosphorylation sites and five kinase families. For example, the models employing the combination of residue-level features and the *context2vec*^*inference*^ (*ws* = 7) improved the average AUC scores by 0.045, 0.025 and 0.005 for phosphorylation site prediction on S, T, and Y sites, and by 0.013, −0.002, 0.126, 0.027, and 0.004 for kinase AGC/PKA, AGC/PKC, CMGC/CDK CMGC/CK2, and TK/SRC, respectively, compared with the models trained with residue-level features only. The only exception was the AGC/PKC kinase, for which the model trained with *context2vec*^*inference*^ of window size 7 performed slightly worse than the baseline model. However, the average AUC achieved with *Residue-level* + *Context2vec*^*inference*^ feature vectors was increased from 0.897 to 0.909 when increasing the contextual window size from 7 to 19, resulting in better performance than that was achieved by models trained with residue-level features only. These improved results demonstrate that, for both general and kinase-specific phosphorylation site prediction, the prediction performance achieved with the *Residue-level* + *Context2vec* features could be further improved when the contextual window sizes of *Context2vec* features were appropriately selected. This was more pronounced for phosphorylation site types S and T, and the CMGC/CDK kinase, with the average AUC scores significantly increased by 0.047, 0.031 and 0.126 (significant level: 5%), respectively.

Comparing the predictive performance achieved by the models trained with *Context2vec-*only and *Residue-level* + *Context2vec* features, it can be observed that the models trained with the latter outperformed those trained with the former (Tables 2 and 3). Taking kinase-specific prediction as an example, the best performance in terms of AUC scores was improved from 0.918, 0.865, 0.890, 0.893, and 0.913 to 0.938, 0.909, 0.925, 0.919, and 0.966 for AGC/PKA, AGC/PKC, CMGC/CDK, CMGC/CK2, and TK/Src, respectively, when *Context2vec*^*inference*^ was used in combination with *Residue-level* features. Similar improvements can be observed by comparing the results achieved by *Context2vec*^*add*^ and *Residue-level* + *Context2vec*^*add*^. For example, the best AUC scores for the five kinase families were improved from 0.925, 0.885, 0.903, 0.901, and 0.931 to 0.940, 0.915, 0.907, 0.929, and 0.964, respectively, when *Context2vec*^*add*^ was used in combination with *Residue-level* features. The results suggest that the *Context2vec*, as a useful contextual feature vector, can achieve better predictive performance when used in combination with residue-level features.

In terms of the effect of contextual window size, the performance (evaluated in terms of AUC scores) seemed to depend on the phosphorylation site type and kinase family. In general, with the increase of the contextual window size, the AUC score tended to increase until reaching the peak and then started to decrease. For example, the average AUC score of the models trained using *Residue-level* + *Context2vec* features was improved from 0.887 to 0.892 and decreased to 0.891 when the contextual window size was increased from 7 to 15 and decreased from 15 to 19, respectively, for S phosphorylation site prediction. For CMGC/CDK, the best AUC score of 0.926 was achieved using the contextual window size of 7, and then decreased to 0.899 when the window size was increased to 19. As aforementioned, *Residue-level* features can be seen as the special case where the contextual window size is set as 0, whereas the sequence-level representation *prot2vec*^*add*^ (i.e. *ProtVec*) is equivalent to the special case where the contextual window size is set as infinite. According to the results in Tables 2 and 3, models that were trained with these two types of feature vectors achieved worse performance compared to that of the models trained with *Context2vec* features. These results highlight the need and importance of developing specialized distributed representation of local sequence contexts that are more informative to address the residue-level prediction tasks, such as phosphorylation site prediction in this study.

### Comparison between the two implementation strategies of Context2vec

**Figure 3:**
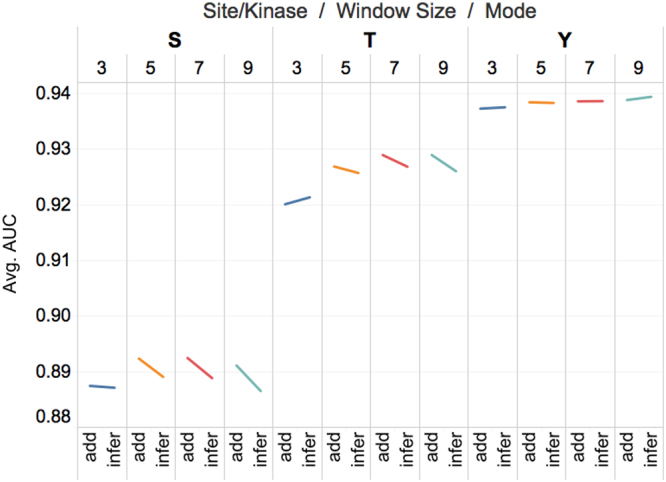
Performance comparison between the models trained based on *context2vec*^*add*^ and *context2vec*^*inference*^ feature vectors for general phosphorylation site predictions, evaluated in terms of the AUC score. evaluated in terms of the AUC score.

**Figure 4:**
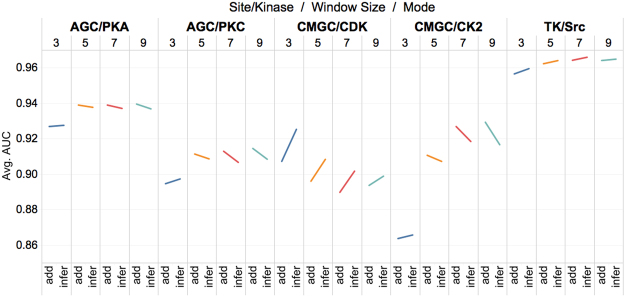
Performance comparison between the models trained based on *context2vec*^*add*^ and *context2vec*^*inference*^ feature vectors for kinase-specific phosphorylation site predictions, evaluated in terms of the AUC score. evaluated in terms of the AUC score.

We further compared the predictive performance of models trained using the two implementation strategies *context2vec*^*add*^ and *context2vec*^*inference*^, respectively, based on the same group of results. Figures 3 and 4 plot the average AUC scores achieved by models trained with *context2vec*^*add*^ and *context2vec*^*inference*^ for both general and kinase-specific phosphorylation site predictions. Models trained using different contextual window sizes 0, 7, 11, 15, and 19 (denoted as None, w3, w5, w7, and w9, respectively) are indicated by different colours. For each short line, the left side indicates the average AUC score achieved by the *context2vec*^*add*^ model, while the right side indicates the average AUC score achieved by *context2vec*^*inference*^ model. The tilt angle of each short line thus indicates the performance difference between the two types of contextual feature vectors-based models.

According to Figs 3 and 4, two important observations can be made. The first observation is that, regardless of the selection of the contextual window size, one of the two implementations performed better than the other depending on the type of phosphorylation sites and kinase families. Specifically, in the case of S/T phosphorylation sites and most the of five kinases that phosphorylate S/T phosphorylation sites, models trained with *context2vec*^*add*^ performed better than those trained with *context2vec*^*inference*^, while in the case of Y phosphorylation sites, the TK/SRC kinase that phosphorylates Y sites, and the CMGC/CDK kinase that phosphorylates S/T sites, models trained with *context2vec*^*inference*^ performed better than those trained with *context2vec*^*add*^.

The second observation is that the performance of *context2vec*^*add*^ improved faster than that of the *context2vec*^*inference*^ with the increase of the contextual window size. This is manifested by the relative change of the tilt angle with respect to different contextual window size in Figs 3 and 4. For example, for CMGC/CK2 (represented by the skyblue lines in Fig. 4), the tilt angle had a transition from slightly favoring *context2vec*^*inference*^ to gradually rising up on the side of *context2vec*^*add*^ with the increase of the contextual window size. For CMGC/CDK, the performance of both *context2vec*^*add*^ and *context2vec*^*inference*^ decreased with the increase of the contextual window size, but the performance of *context2vec*^*inference*^ decreased faster than that of *context2vec*^*add*^, resulting in shrinking of the tilt angle with the increase of the contextual window size. The performance difference between the two implementations of *context2vecs* can be better explained by the advantages and disadvantages of *context2vec*^*add*^ and *context2vec*^*inference*^ (Refer to the Introduction section).

According to the AUC scores and their standard deviations shown in Tables 2 and 3, Figs 3 and 4, we finally selected *context2vec*^*add*^ with the window size of 15, *context2vec*^*add*^ with the window size of 15, and *context2vec*^*inference*^ with the window size of 19 for predicting S, T, and Y phosphorylation sites, respectively, and *context2vec*^*add*^ with the window size of 19, *context2vec*^*add*^ with the window size of 19, *context2vec*^*inference*^ with the window size of 7, *context2vec*^*add*^ with the window size of 19, and *context2vec*^*inference*^ with the window size of 15 for predicting phosphorylation sites of AGC/PKA, AGC/PKC, CMGC/CDK, CMGC/CK2 and TK/SRC kinases, respectively. The results suggest that this new distributed representation of contextual features improved the prediction of both general and kinase-specific phosphorylation sites. According to the 10-fold cross-validation results, the overall predictive performance was improved by 0.051, 0.033, and 0.006 for the prediction of S, T, and Y phosphorylation sites, and improved by 0.025, 0.017, 0.127, 0.009, and 0.001 for the prediction of AGC/PKA, AGC/PKC, CMGC/CDK, CMGC/CK2, and TK/SRC phosphorylation sites, respectively, compared with those of the models that were trained with residue-level feature only.

### Determination of hyper-parameters

We trained independent SVM models for each of the three phosphorylation site types and five kinase families, for which two hyper-parameters could influence the predictive performance and hence needed to be determined. The two hyper-parameters were the kernel type, which determined the kernel that was used for training the model and the penalty parameter *C* of the error term that affected the trade-off between the complexity and proportion of non-separable examples^38^. We tested the performance of the models trained with two most commonly used kernel types including the *linear* kernel and the *rbf* kernel (*rbf* was the default kernel), as well as five different values (including 1.0, 3.0, 5.0, 7.0, and 9.0; 1.0 was the default value) of the penalty parameter *C*. Note that a larger penalty *C* indicates a stronger penalty on non-separable examples, which will result in more complex models that fit the data more strictly. However, it may also cause overfitting at the same time. Based on the optimal contextual window sizes and *context2vec* implementation strategy selected from the aforementioned experiments, we performed 10-fold cross-validation tests to compare the performance of models trained with different kernel types and values of penalty parameter *C*. The corresponding results are shown in Figure S1.

As we can see from Figure S1, for general phosphorylation site prediction, the *rbf* kernel performed better for S phosphorylation sites, while the *linear* kernel performed better for T and Y phosphorylation sites. The best performance for S, T, and Y phosphorylation site prediction was achieved when the value of the penalty parameter *C* was set to 3.0, 7.0, and 7.0, respectively. For kinase-specific phosphorylation site prediction, the *rbf* kernel clearly performed better than the *linear* kernel across all the five kinase families. Accordingly, the best performance was achieved when the values of *C* were set to 5.0, 9.0, 9.0, 3.0 and 3.0 for the AGC/PKA, AGC/PKC, CMGC/CDK, CMGC/CK2, and TK/SRC kinase families, respectively.

### General phosphorylation site prediction performance in independent tests

**Figure 5:**
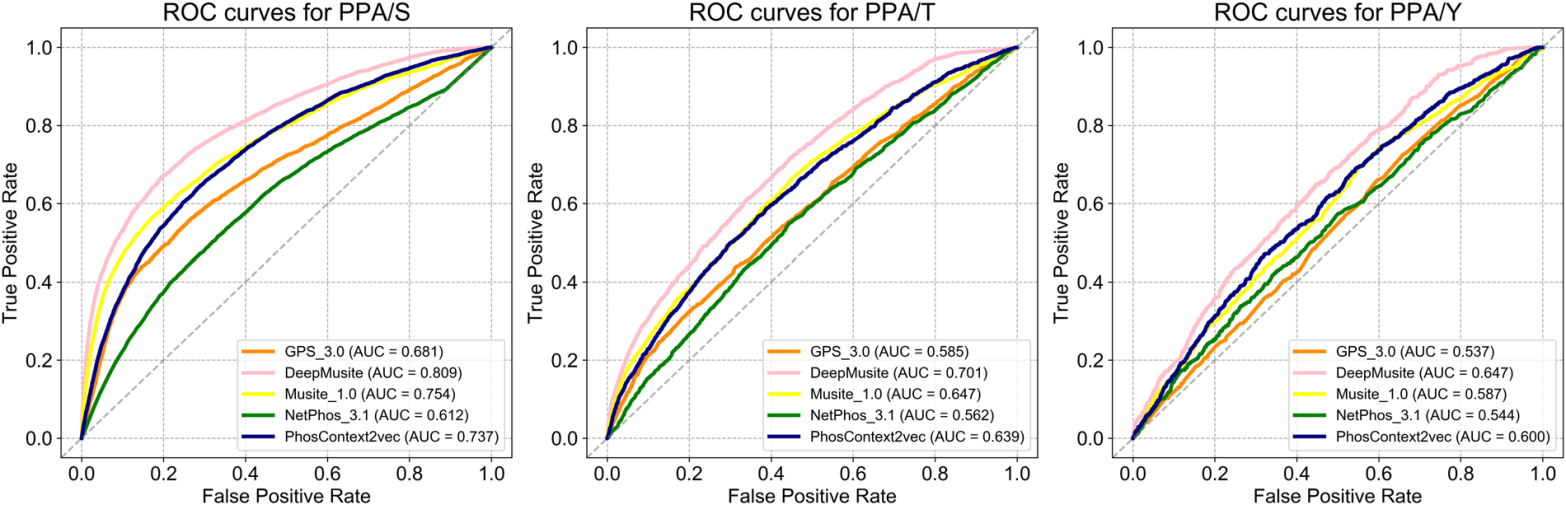
ROC curves of PhosContext2Vec and four other methods on the independent test for general phosphorylation site prediction. The ROC curves of different methods are indicated by different colours. An AUC score of 0.5 indicates a random prediction.

In order to validate the performance of PhosContext2vec for general phosphorylation site prediction, we further performed independent tests. Using the optimized contextual window size, *context2vec* representation implementations and hyper-parameters selected on the cross-validation, we trained the models for S, T, and Y sites using the PELM training datasets and then evaluated the performance using the curated PPA datasets. To compare the performance of PhosContext2vec with other existing methods, we submitted the same datasets to the other predictors including GPS 3.0, MusiteDeep, Musite 1.0, and NetPhos 3.1 and collected the corresponding prediction results. Figure 5 shows the ROC curves and AUC scores of all the compared methods for general phosphorylation site prediction.

According to the results shown in Fig. 5, MusiteDeep achieved the best predictive performance in terms of AUC scores amongst all the compared methods for general phosphorylation site prediction. In particular, MusiteDeep achieved an overall best performance for S, T and Y phosphorylation site prediction with an AUC score of 0.809, 0.701, and 0.647, respectively, while Musite 1.0 achieved the second-best AUC scores of 0.754 and 0.647 for S and T phosphorylation site prediction and PhosContext2vec achieved the second-best AUC score of 0.600 for Y phosphorylation site prediction. PhosContext2vec achieved the third-best performance for S and T phosphorylation site prediction with an average AUC score of 0.737 and 0.639, respectively.

Among the five compared methods, MusiteDeep is the only method that was developed based on the deep learning architecture. It used raw amino acid sequence as the input, extracted high abstract representations from a peptide of 33 residues centered around a potential phosphorylation site using convolutional layers and the attention mechanism, and performed phosphorylation site prediction based on the extracted representations^35^. In comparison, PhosContext2vec was developed based on a traditional way of decoupling the process of feature extraction and model training. The SVM algorithm used has a comparatively less expressive power^39^, which may partially explain the inferior performance of PhosContext2vec compared to that of MusiteDeep. Nevertheless, the representation extracted by MusiteDeep purely rely on the neighboring residues of the target residue, while the contextual feature vectors used by PhosContext2vec also involve the information that is distributed in large protein sequence databases, thereby presenting some advantages in terms of contextual feature vector representation.

Compared with GPS 3.0 and NetPhos 3.1, both Musite 1.0 and PhosContext2vec considered protein disorder information as the input feature for training the models. In addition, Musite 1.0 also considered amino acid frequencies and used an ensemble learning strategy to perform the final prediction, which might be superior to individual SVM models or neural network models. GPS 3.0 was designed to predict phosphorylation sites in a way that kinases were clustered in a hierarchical structure, where the training samples of other related kinases can be reused for training better models. In the case of general phosphorylation site prediction, where models were respectively trained for S, T, and Y sites (i.e. not in a hierarchical manner), the hierarchical clustering of kinases did not appear to help improve the predictive performance. NetPhos 3.1 predicted phosphorylation sites by exploiting local sequential patterns in combination with neural network learning. However, different from NetPhos 3.1, PhosContext2vec incorporated both residue-level and contextual-level feature vectors in an integrated manner. In addition, the contextual feature vectors were generated from a number of different contextual patterns present in large protein sequence databases, which might explain why PhosContext2vec achieved a better performance compared to NetPhos 3.1.

Among the three types of phosphorylation sites, we found that the performance of the S site was better than that of the T site, while the performance of Y site was the worst. According to a previous study^22^, PROSITE motifs could only recognize 10% of annotated Y phosphorylation sites^40^, while being able to recognize 48% and 38% of the respective S and T phosphorylation sites. This indicates that the local patterns of phosphorylated tyrosine sites are much more difficult to capture, making them more challenging to be predicted.

### Kinase-specific phosphorylation site prediction performance in independent tests

**Figure 6:**
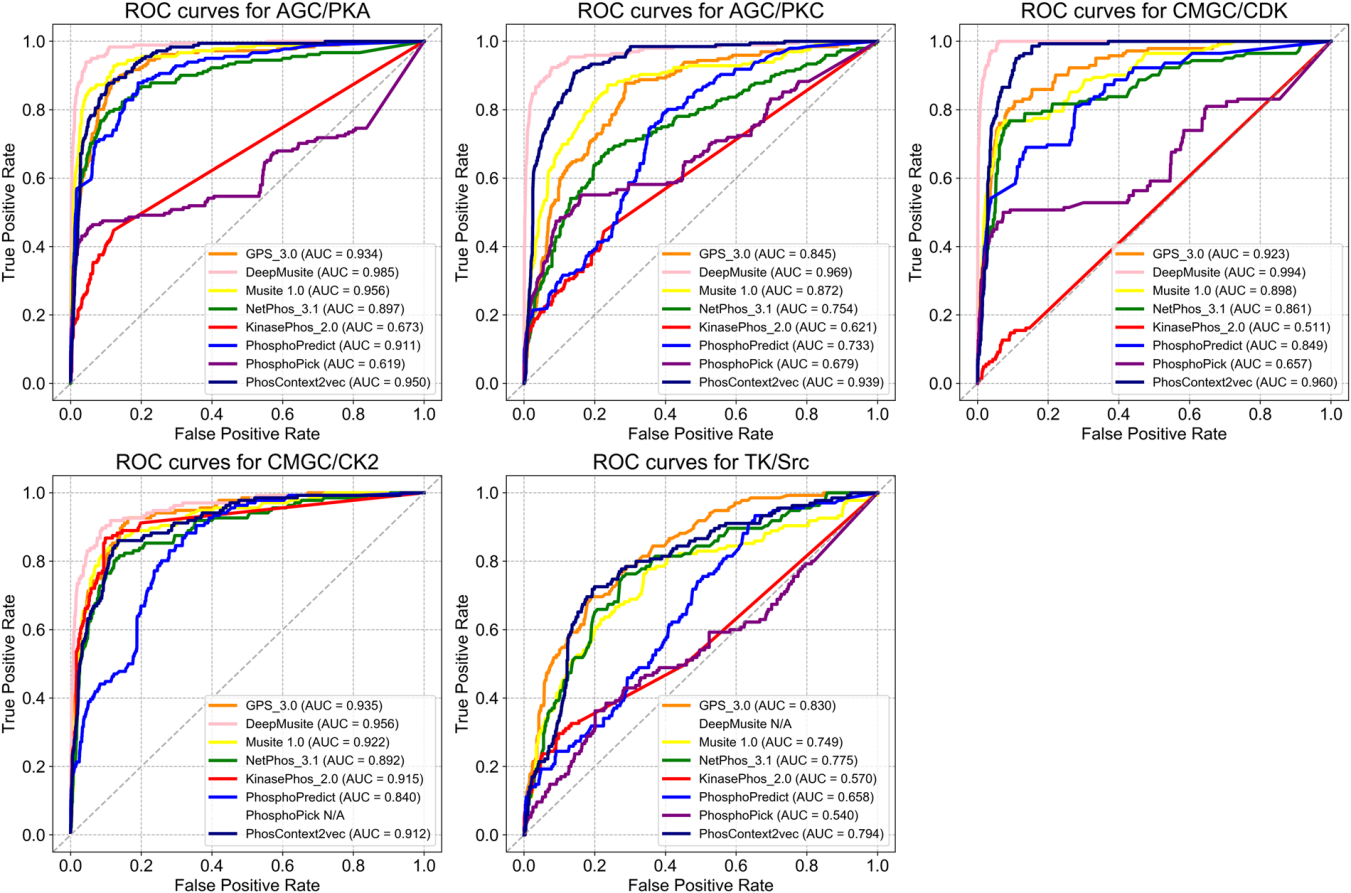
ROC curves of PhosContext2vec and seven other existing methods for kinase-specific phosphorylation site prediction on the independent test. The ROC curves of different methods are indicated by different colours. Five panels correspond to the prediction results of AGC/PKA, AGC/PKC, CMGC/CDK, CMGC/CK2, and TK/Src kinase families, respectively. The performance of a method was denoted as “*N/A*” if such method does not provide pre-trained models to predict the phosphorylation sites for the corresponding kinase family.

In this section, in order to validate the performance of PhosContext2vec and compare it performance with other methods for kinase-specific phosphorylation site prediction, we further performed an independent test using the testing datasets extracted from UniProt and Phospho.ELM (Refer to the Methods section). The compared models include GPS 3.0, MusiteDeep, Musite 1.0, NetPhos 3.1, KinasePhos 2.0 PhosphoPredict, and PhosphoPick. Figure 6 shows the ROC curves and the AUC scores of these methods.

As shown in Fig. 6, different methods achieved varying performance for the five tested kinase families. Among all the compared methods, the most recently developed MusiteDeep method outperformed all other methods, demonstrating an outstanding performance across all the four tested kinase families, with an AUC score ranging between 0.934 and 0.973. For AGC/PKA, MusiteDeep achieved the best AUC score of 0.976, while Musite 1.0 performed the second-best with an average AUC of 0.956, followed by PhosContext2vec which achieved an AUC of 0.950. GPS 3.0 achieved an AUC score of 0.934, while PhosphoPredict achieved an AUC score of 0.911. Compared to the five other predictors that achieved an AUC score of >0.90, KinasePhos 2.0 and PhosphoPick achieved the worst AUC scores in a range of between 0.6 and 0.7.

For AGC/PKC and CMGC/CDK, PhosContext2vec achieved the second-best AUC scores of 0.939 and 0.960, respectively, secondary to MusiteDeep which achieved an AUC score of 0.954 and 0.973, respectively. Musite 1.0 and GPS 3.0 also performed relatively well compared with the rest of the benchmarked predictors. For CMGC/CK2, PhosphoPick could not predict the potential phosphorylation sites for this kinase family, while all the other predictor achieved a similar performance. Among these predictors, MusiteDeep achieved the best AUC score of 0.950 and PhosphoPredict achieved the lowest AUC score of 0.840, respectively. Finally, for TK/Src, GPS 3.0 achieved the best performance with an AUC score of 0.830, while PhosContext2vec achieved the second-best performance with an AUC score of 0.794. Overall, MusiteDeep, Musite 1.0, GPS 3.0 and PhosContext2vec were evaluated as the top four best-performing predictors among all the compared methods, while PhosphoPick and KinasePhos 2.0 performed the worst in the independent test.

The inferior performance of KinasePhos 2.0 may be explained partly by the incomplete results obtained from its web server. The current web server of KinasePhos 2.0 only predicted phosphorylation sites with scores above the given specificity thresholds (four options are available: default, 80%, 90% and 100%). NetPhos 3.1 performed well for CMGC/CK2 and TK/Src and relatively well for other kinase families, for example AGC/PKA. PhosphoPick achieved inferior performance for most tested kinases. It is the only predictor developed based on integrating protein functional features such as protein-protein interactions. However, on the other hand, it did not consider any features from amino acid sequences^37^. We would recommend that it should be used in combination with other sequence-based predictors to achieve a better performance. To quantify the performance of different predictors in terms of other measurements, we also calculated sensitivity, specificity, and MCC values. As mentioned in the Performance evaluation section, the low, medium and high cut-off FPRs for S/T sites were set to 2%, 6% and 10%, while the low, medium and high cut-off FPRs for T sites were set to 4%, 9% and 15%, respectively. In Tables S3 and S4, we used the same cut-off FPRs for comparing the different predictors.

It is noteworthy that MusiteDeep represents the only method available to date that employed the deep learning technique. We would like to point out that its superior performance over all other methods was consistent despite of its use of raw protein sequence as the direct input to train the deep learning models^35^. As an effective contextual feature vector, we hope that PhosContext2vec can be used as a side channel^41^ to further improve the performance of deep learning frameworks such as MusiteDeep in future work.

### Comparison between different methods in terms of time efficiency

To better understand the processing efficiency of different methods, we measured the elapsed time for processing the same 200 protein sequences using each predictor. The five compared predictors included GPS 3.0, Musite 1.0, MusiteDeep, NetPhos 3.1, KinasePhos 2.0, and PhosphoPick. For these predictors, we tested the standalone Java programs of the former two, the python source codes of MusiteDeep, and the online web servers of the latter three. For PhosContext2vec, we tested both the standalone CPU time and the response time of the web server.

**Figure 7:**
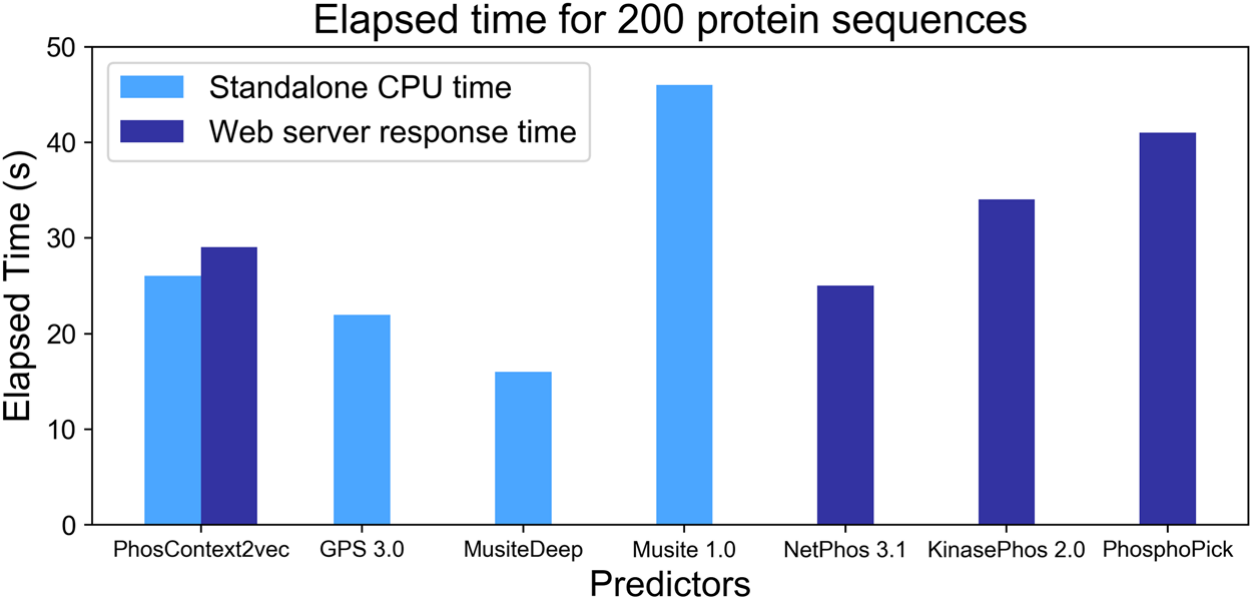
Elapsed time comparison between different phosphorylation site predictors for predicting 200 protein sequences. Due to the limited availability of the external predictors, we calculated the CPU time (denoted by light blue) for those predictors with available standalone programme. For those predictors with only web servers available, we measured their response time (denoted by dark blue). For PhosContext2vec, both the CPU time and the web server response time was provided.

As shown in Fig. 7, among the four tools PhosContext2vec, GPS 3.0, MusiteDeep, and Musite 1.0 that were evaluated in terms of the CPU time, MusiteDeep provided the fastest computing speed and completed the task within 16 seconds. In particular, the CPU time of Musite 1.0 was calculated given that protein disorder information has been cached. Similarly, the CPU time of PhosContext2vec was calculated on the assumption that the BLAST results were cached. It took Musite 1.0~45 seconds to process 200 protein sequences, while it took PhosContext2vec about 25 seconds to complete the same prediction task. In terms of the web server response time, NetPhos 3.1 benefited from the use of multiple threads and achieved the fastest speed within 25 seconds. As a comparison, it took KinasePhos 2.0 and PhosphoPick ~35 seconds and more than 40 seconds, respectively, for processing the same 200 sequences. It took the PhosContext2vec web server approximately 28 seconds to complete the prediction of 200 protein sequences.

### Implementation of the PhosContext2vec web server

The PhosContext2vec web server is currently configured and hosted on a virtual server machine deployed in the Monash e-Research Centre at Monash University, equipped with four cores, 12 GB memory and a 110 GB hard disk. It is implemented using the Python-Django framework^42^ and consists of three major components, i.e. the client interface, the backend server, and the asynchronous task scheduler. The user interface interacts with the clients, collects protein sequence inputs, parameter settings, and email addresses and forwards the submitted requests to the backend server. The backend server interacts with the client interface and determines the logic of each request. Following the submission of each request, the backend server starts a running job and forwards the generated results to the client interface once the job is completed. For long-running jobs, we employed a third component, which is an asynchronous task scheduler, for optimizing the allocation of computational resources. With this architecture, the backend server can return a real-time task status before the task is completed, which sets the server threads free to deal with more requests. Figure S2 shows the architecture of the PhosContext2vec web server.

The PhosContext2vec web server provides three different but complementary functions for users, including contextual feature vector generation, general phosphorylation site prediction and kinase-specific phosphorylation site prediction. Users can submit their protein sequences of interest through either entering the sequence information in the text area provided at the web page, or uploading the sequence file in the FASTA format, or supplying a concise list of UniProt IDs of query proteins. In terms of contextual vector generation, four contextual window sizes (i.e. 7, 11, 15, and 19) are available as options. For general and phosphorylation site prediction, the contextual window sizes for different types of phosphorylation sites (including S, T and Y) and kinase families (including AGC/PKA, AGC/PKC, CMGC/CDK, CMGC/CK2, and TK/Src) were set to the optimal values based on the empirical results. Users are allowed to adjust the prediction *cut-off* and *output* thresholds for generating customized prediction results in order to meet their specific requirements. Potential phosphorylation sites predicted with scores larger than the *output* threshold will be included in the prediction output; however, only those with scores larger than the prediction *cut-off* threshold will be considered as a positive prediction. On task completion, users can download the text-based results, browse the web page-based result summaries and visualize the graphical statistics. Alternatively, for users who provide their email address, an email with the output links and file attachments will be sent. Detailed examples of using the PhosContext2vec web server and descriptions of the generated outputs are provided in Figures S3 and S4 in the Supplementary Material.

## Discussions

In this study, we have proposed an effective distributed representation of contextual feature vectors for general and kinase-specific phosphorylation site prediction. Compared to previous contextual patterns for potential phosphorylation sites^3,5,9,21^ the distributed contextual feature vector has several attractive advantages. First, the distributed representation is automatically generated from a pre-trained feature extraction model that synthesizes patterns from all possible contexts in large protein sequence databases; Second, the implementation of contextual feature vector comes in two complementary flavors where *context2vec*^*add*^ has higher accuracies for individual biological words while *context2vec*^*inference*^ additionally models the internal order of the biological words; Third, the resulting contextual feature vector is represented as a one-dimensional real-valued vector with fixed vector size, thereby making it more convenient to be used in combination with other feature vectors; Finally, such contextual feature vector can be used for any residue-level protein property prediction that fully or partially relies on extraction of local sequence contexts, for example, protein secondary structure prediction^43,44^, protein disorder prediction^15,45^, protein torsion angle prediction^46,47^ as well as other types of protein PTM sites^26,44^ and protease cleavage sites^29,48^.

Based on the distributed contextual representation, we applied the contextual feature vectors to solve the prediction problems of both general and kinase-specific phosphorylation sites. We conducted cross-validation tests for optimizing the selection of contextual window sizes, contextual representation implementations, and hyper-parameters. Improved performance was achieved for all the three types of phosphorylation sites and five kinase families when the contextual feature vector was incorporated. When evaluated on the independent test and compared to several other state-of-the-art predictors, the developed PhosContext2vec model based on the distributed contextual feature vector achieved a relatively superior performance for predicting Y phosphorylation sites, and also for the AGC/PKC and CMGC/CDK kinase families. It also achieved promising performance for S and T phosphorylation sites, and for AGC/PKA and TK/SRC kinase families.

As for kinase-specific phosphorylation site prediction, the PhosContext2vec online webserver was designed to predict the potential phosphorylation sites of the 138 kinase groups, families, subfamilies, and protein kinases arranged at different hierarchical levels; however, we only performed benchmarking tests for five kinases that had more than 500 experimentally validated phosphorylation sites. In future work, more kinases will be included in the online web server of PhosContext2vec when more experimentally validated phosphorylation data become available for such kinases.

## Electronic supplementary material

SI
