# Supplementary material for: PhosContext2vec: a distributed representation of residue-level sequence contexts and its application to general and kinase-specific phosphorylation site prediction
Source: Sci Rep. 2018 May 29;8:8240. doi: 10.1038/s41598-018-26392-7 (PMC5974293; doi:10.1038/s41598-018-26392-7)
Supplement: Supplementary file 1 — SI [file 41598_2018_26392_MOESM1_ESM.docx]

**PhosContext2vec: a distributed representation of residue-level sequence contexts and its application to general and kinase-specific phosphorylation site prediction**

Ying Xu^1,2^, Jiangning Song^2,3,4,*^, Campbell Wilson^1,*^, James C. Whisstock^3,4^

^1^Faculty of Information Technology, Monash University, Melbourne, VIC 3800, Australia

^2^Monash Centre for Data Science, Faculty of Information Technology, Monash University, Melbourne, VIC 3800, Australia

^3^Infection and Immunity Program, Biomedicine Discovery Institute and Department of Biochemistry and Molecular Biology, Monash University, Melbourne, VIC 3800, Australia

^4^ARC Centre of Excellence in Advanced Molecular Imaging, Monash University, Melbourne, VIC 3800, Australia

**Supplementary Information**

**SUPPLEMENTARY TABLES**

**Table S1** and **Table S2** provides a statistical summary of constructed datasets for kinase-specific phosphorylation site prediction. The kinases are clustered as a multi-level hierarchical structure as previously described in GPS 2.0 [1]. This hierarchical structure is composed of four levels including groups, families, subfamilies and kinases. As mentioned in the main manuscript, we constructed the datasets for kinase-specific phosphorylation site prediction by combining annotations extracted from the UniProt [2] and the Phospho.ELM databases [3].

In **Table S1**, we demonstrated the dataset statistics of the five reported protein families, including AGC/PKA, AGC/PKC, CMGC/CDK, Other/CK2, and TK/Src, in cross-validation and independent tests. According to the manuscript, the cross-validation tests were performed on the training datasets while the independent tests were conducted on the testing datasets. In cross-validation tests, an equal number of negative samples with the number of positive samples were randomly selected for training the model, with a ratio of 1:1 between the number of positives and negatives. For each of the five protein kinase families, we provided the total number of negative samples (S, T, and Y sites that are not annotated as phosphosites) from which the down-sampling was performed in **Table S1**. In independent test, we did not perform down-sampling, but only included S, T or Y sites that are phosphorylated by specific kinases as the negative samples. For AGC/PKA, AGC/PKC, CMGC and Other/CK2, we included all S and T sites that are not annotated as phosphosites as the negative samples. For TK/Src we included all Y sites that are not annotated as phosphosites as the negative samples. Therefore, the number of negative sample in the testing datasets of AGC/PKA, AGC/PKC, CMGC/CDK, Other/CK2, and TK/Src in **Table S1** are different from those in **Table S2** which include S, T, and Y sites that are not annotated as phosphosites.

**Table S1. Statistical summary of constructed datasets for the five reported kinase families.**

|  | **AGC/PKA** | | **AGC/PKC** | | **CMGC/CDK** | | **Other/CK2** | | **TK/Src** | |
| --- | --- | --- | --- | --- | --- | --- | --- | --- | --- | --- |
|  | *#pos* | *#neg* | *#pos* | *#neg* | *#pos* | *#neg* | *#pos* | *#neg* | *#pos* | *#neg* |
| **Training** | 716 | 58,967 | 766 | 41,473 | 486 | 34,192 | 532 | 20,564 | 496 | 29,591 |
| **Testing** | 181 | 15,288 | 196 | 10,672 | 142 | 7,443 | 136 | 6,167 | 135 | 8,246 |

In **Table S2**, each line corresponds to a kinase (including kinase groups, families, subfamilies and protein kinases) and the number of positive and negative samples in its constructed training and testing datasets. The negative samples for each kinase include the S, T, and Y sites that are not annotated as phosphorylation sites. The kinase is denoted by its path from the root ‘group’ in the hierarchical structure, thereby resulting in the path format of *group/family/subfamily/kinase*.

**Table S2. Statistical summary of constructed datasets for the 137 kinase groups, families, subfamilies and protein kinases.**

|  | #pos | #neg |  |  |
| --- | --- | --- | --- | --- |
| AGC | 2,090 | 124,479 | 518 | 31,953 |
| AGC/DMPK | 99 | 5,107 | 26 | 1,515 |
| AGC/DMPK/ROCK | 88 | 4,918 | 30 | 1,548 |
| AGC/DMPK/ROCK/ROCK1 | 36 | 2,057 | 18 | 783 |
| AGC/DMPK/ROCK/ROCK2 | 43 | 2,955 | 11 | 1,036 |
| AGC/GRK | 254 | 5,457 | 54 | 1,400 |
| AGC/GRK/BARK | 150 | 3,344 | 35 | 1,086 |
| AGC/GRK/BARK/GRK2 | 131 | 2,846 | 42 | 909 |
| AGC/GRK/GRK | 108 | 2,237 | 25 | 465 |
| AGC/GRK/GRK/GRK6 | 58 | 984 | 11 | 772 |
| **AGC/PKA** | **716** | **58,967** | **181** | **15,288** |
| AGC/PKA/- | 472 | 42,251 | 132 | 12,185 |
| AGC/PKA/-/PKA | 472 | 42,251 | 132 | 12,185 |
| AGC/PKB | 256 | 20,270 | 60 | 5,784 |
| AGC/PKB/- | 52 | 1,977 | 11 | 783 |
| AGC/PKB/-/PDK1 | 52 | 1,977 | 11 | 783 |
| **AGC/PKC** | **766** | **41,473** | **196** | **10,672** |
| AGC/PKC/Alpha | 158 | 9,979 | 54 | 3,239 |
| AGC/PKC/Alpha/PKCA | 157 | 9,705 | 39 | 2,221 |
| AGC/PKC/Delta | 51 | 3,878 | 12 | 1,590 |
| AGC/PKC/Delta/PKCD | 51 | 3,878 | 12 | 1,590 |
| AGC/PKC/Eta | 41 | 1,861 | 9 | 1,028 |
| AGC/PKC/Eta/PKCE | 41 | 1,861 | 9 | 1,028 |
| AGC/PKC/Iota | 36 | 3,473 | 11 | 1,010 |
| AGC/PKC/Iota/PKCZ | 33 | 3,003 | 9 | 808 |
| AGC/PKG | 72 | 7,061 | 17 | 1,476 |
| AGC/RSK | 52 | 3,841 | 14 | 1,123 |
| AGC/RSK/RSK | 40 | 3,357 | 9 | 573 |
| AGC/SGK | 70 | 5,286 | 35 | 1,712 |
| AGC/SGK/- | 57 | 4,271 | 28 | 1,379 |
| AGC/SGK/-/SGK1 | 61 | 4,024 | 18 | 1,017 |
| Atypical | 182 | 12,016 | 42 | 3,191 |
| Atypical/PIKK | 183 | 12,047 | 37 | 3,043 |
| Atypical/PIKK/ATM | 151 | 10,183 | 38 | 3,066 |
| Atypical/PIKK/ATM/ATM | 151 | 10,183 | 38 | 3,066 |
| Atypical/PIKK/ATR | 34 | 2,168 | 22 | 1,034 |
| Atypical/PIKK/ATR/ATR | 34 | 2,168 | 22 | 1,034 |
| CAMK | 778 | 50,519 | 171 | 13,955 |
| CAMK/CAMK1 | 68 | 4,568 | 17 | 2,065 |
| CAMK/CAMK1/- | 28 | 2,120 | 9 | 848 |
| CAMK/CAMK1/-/CAMK4 | 17 | 1,522 | 11 | 951 |
| CAMK/CAMK2 | 128 | 10,067 | 34 | 4,546 |
| CAMK/CAMK2/- | 50 | 4,660 | 11 | 2,174 |
| CAMK/CAMK2/-/CAMK2A | 27 | 2,725 | 13 | 605 |
| CAMK/CAMKL | 316 | 28,251 | 82 | 6,757 |
| CAMK/CAMKL/AMPK | 168 | 16,562 | 49 | 4,132 |
| CAMK/CAMKL/BRSK | 25 | 1,607 | 8 | 593 |
| CAMK/CAMKL/LKB | 36 | 4,233 | 12 | 1,275 |
| CAMK/CAMKL/LKB/LKB1 | 36 | 4,233 | 12 | 1,275 |
| CAMK/CAMKL/MARK | 35 | 3,650 | 12 | 1,391 |
| CAMK/CAMKL/MARK/MARK1 | 18 | 2,413 | 9 | 635 |
| CAMK/CAMKL/NuaK | 24 | 1,692 | 10 | 925 |
| CAMK/CAMKL/NuaK/NUAK1 | 24 | 1,692 | 10 | 925 |
| CAMK/DAPK | 44 | 2,274 | 9 | 310 |
| CAMK/DAPK/- | 44 | 2,251 | 8 | 288 |
| CAMK/DAPK/-/DAPK1 | 28 | 1,833 | 9 | 262 |
| CAMK/MAPKAPK | 85 | 5,051 | 34 | 1,296 |
| CAMK/MAPKAPK/MAPKAPK | 95 | 4,660 | 21 | 1,497 |
| CAMK/MAPKAPK/MAPKAPK/MAPKAPK2 | 72 | 3,416 | 18 | 1,036 |
| CAMK/MAPKAPK/MAPKAPK/MAPKAPK5 | 23 | 1,205 | 14 | 755 |
| CAMK/PHK | 74 | 2,747 | 27 | 1,145 |
| CAMK/PKD | 50 | 3,283 | 11 | 890 |
| CK1 | 229 | 11,667 | 70 | 2,944 |
| CK1/CK1 | 214 | 11,924 | 58 | 1,969 |
| CK1/VRK | 29 | 1,554 | 14 | 660 |
| CK1/VRK/- | 29 | 1,554 | 14 | 660 |
| CK1/VRK/-/VRK1 | 29 | 1,554 | 14 | 660 |
| CK1/VRK/-/VRK2 | 24 | 1,239 | 8 | 433 |
| CMGC | 936 | 58,500 | 263 | 13,783 |
| **CMGC/CDK** | **486** | **34,192** | **142** | **7,443** |
| CMGC/CDK/CDC2 | 215 | 14,529 | 47 | 3,713 |
| CMGC/CDK/CDC2/CDK2 | 182 | 14,731 | 51 | 2,721 |
| CMGC/CDK/CDK5 | 159 | 13,219 | 42 | 3,384 |
| CMGC/CDK/CDK5/CDK5 | 159 | 13,219 | 42 | 3,384 |
| CMGC/CDK/CDK7 | 37 | 1,979 | 7 | 648 |
| CMGC/CDK/CDK7/CDK7 | 37 | 1,979 | 7 | 648 |
| CMGC/CDK/CDK9 | 39 | 2,251 | 5 | 641 |
| CMGC/CDK/CDK9/CDK9 | 39 | 2,251 | 5 | 641 |
| CMGC/DYRK | 144 | 9,503 | 37 | 2,775 |
| CMGC/DYRK/Dyrk1 | 17 | 1,554 | 4 | 368 |
| CMGC/DYRK/Dyrk2 | 80 | 6,529 | 20 | 1,677 |
| CMGC/DYRK/Dyrk2/DYRK2 | 70 | 6,181 | 26 | 1,667 |
| CMGC/DYRK/HIPK | 60 | 2,779 | 17 | 597 |
| CMGC/DYRK/HIPK/HIPK2 | 49 | 2,417 | 13 | 596 |
| CMGC/GSK | 212 | 15,101 | 59 | 3,372 |
| CMGC/GSK/GSK3B | 212 | 15,101 | 59 | 3,372 |
| CMGC/MAPK | 158 | 11,054 | 42 | 2,382 |
| Other | 894 | 43,866 | 237 | 11,192 |
| **Other/CK2** | **532** | **20,564** | **136** | **6,167** |
| Other/CK2/- | 100 | 5,697 | 22 | 1,197 |
| Other/CK2/-/CK2A | 100 | 5,697 | 22 | 1,197 |
| Other/IKK | 80 | 5,824 | 52 | 2,025 |
| Other/IKK/- | 83 | 6,173 | 22 | 1,436 |
| Other/IKK/-/IKKB | 38 | 1,907 | 14 | 1,117 |
| Other/IKK/-/IKKE | 18 | 1,162 | 7 | 360 |
| Other/IKK/-/TBK1 | 25 | 1,567 | 10 | 479 |
| Other/NEK | 36 | 1,846 | 8 | 629 |
| Other/NEK/- | 36 | 1,846 | 8 | 629 |
| Other/PLK | 197 | 15,489 | 64 | 3,857 |
| Other/PLK/- | 197 | 15,489 | 64 | 3,857 |
| Other/PLK/-/PLK1 | 108 | 8,932 | 30 | 1,980 |
| Other/PLK/-/PLK3 | 51 | 3,565 | 17 | 960 |
| STE | 192 | 9,822 | 49 | 2,764 |
| STE/STE20 | 121 | 6,672 | 27 | 1,372 |
| STE/STE20/PAKA | 99 | 5,072 | 24 | 1,448 |
| STE/STE20/PAKA/PAK1 | 45 | 2,848 | 14 | 1,891 |
| STE/STE20/PAKA/PAK2 | 52 | 1,644 | 14 | 861 |
| STE/STE20/PAKA/PAK3 | 23 | 1,359 | 8 | 418 |
| STE/STE7 | 46 | 1,657 | 20 | 666 |
| STE/STE7/- | 46 | 1,657 | 20 | 666 |
| TK | 1,099 | 54,316 | 265 | 13,254 |
| TK/Abl | 66 | 4,154 | 10 | 783 |
| TK/Abl/- | 66 | 4,154 | 10 | 783 |
| TK/Abl/-/ABL | 66 | 4,154 | 10 | 783 |
| TK/Csk | 116 | 3,919 | 41 | 1,344 |
| TK/Csk/- | 116 | 3,919 | 41 | 1,344 |
| TK/Csk/-/CSK | 116 | 3,919 | 41 | 1,344 |
| TK/EGFR | 73 | 4,178 | 15 | 1,469 |
| TK/EGFR/- | 73 | 4,178 | 15 | 1,469 |
| TK/EGFR/-/EGFR | 73 | 4,178 | 15 | 1,469 |
| TK/InsR | 68 | 2,405 | 32 | 1,440 |
| TK/InsR/- | 68 | 2,405 | 32 | 1,440 |
| TK/InsR/-/INSR | 65 | 2,733 | 16 | 564 |
| TK/JakA | 82 | 5,147 | 14 | 1,126 |
| TK/JakA/- | 82 | 5,147 | 14 | 1,126 |
| TK/JakA/-/JAK2 | 58 | 3,754 | 13 | 999 |
| TK/PDGFR | 43 | 2,549 | 15 | 1,237 |
| **TK/Src** | **496** | **29,591** | **135** | **8,246** |
| TK/Src/- | 494 | 29,678 | 131 | 7,779 |
| TK/Src/-/FYN | 79 | 6,170 | 32 | 1,671 |
| TK/Src/-/LCK | 53 | 2,836 | 21 | 1,440 |
| TK/Src/-/LYN | 70 | 3,465 | 11 | 1,432 |
| TK/Src/-/SRC | 298 | 17,987 | 83 | 5,299 |
| TK/Syk | 74 | 3,172 | 20 | 831 |
| TK/Syk/- | 74 | 3,172 | 20 | 831 |
| TK/Syk/-/SYK | 62 | 2,380 | 17 | 1,140 |
| TK/Tec | 37 | 2,197 | 14 | 1,027 |
| TK/Tec/- | 37 | 2,197 | 14 | 1,027 |

**Tables S3** and **S4** show the performance comparison results of different predictors in general and kinase-specific phosphorylation site prediction, in terms of Sensitivity, Specificity, and Matthew’s coefficients of correlation (MCC). For detailed discussions of these performance measurements, please refer to **Performance Evaluation** section in the main manuscript. We used the FPR cut-off thresholds to determine positive prediction from negative predictions in models output real-valued scores. According to GPS 3.0, the low, medium and high cut-off FPRs for S and T sites were set to 2, 6 and 10%, while the low, medium and high cut-off FPRs for T sites were set to 4, 9, 15% respectively. In this study, we used the same sets of FPR cut-off thresholds for calculating the results reported in **Table S3** and **S4**.

**Table S3**. **Performance comparison between different predictors of general phosphorylation sites, evaluated in terms of Sensitivity (SE), Specificity (SP) and Matthews correlation coefficient (MCC)**.

|  | **Predictor** | **GPS 3.0** | | | **MusiteDeep** | | | **Musite 1.0** | | | **NetPhos 3.1** | | | **PhosContext2vec** | | |
| --- | --- | --- | --- | --- | --- | --- | --- | --- | --- | --- | --- | --- | --- | --- | --- | --- |
| **Datasets** | Cutoff threshold | SE | SP | MCC | SE | SP | MCC | SE | SP | MCC | SE | SP | MCC | SE | SP | MCC |
| **PPA.S** | Low | 9.0 | 98.0 | 8.2 | **28.8** | 98.0 | **28.0** | 20.8 | 98.0 | 20.6 | 3.0 | 98.9 | 2.9 | 11.2 | 98.0 | 10.6 |
|  | Medium | 25.5 | 94.0 | 13.6 | **45.2** | 94.0 | **26.2** | 38.5 | 94.1 | 22.2 | 12.0 | 95.3 | 5.9 | 27.1 | 94.0 | 14.7 |
|  | High | 36.6 | 90.0 | 14.8 | **53.2** | 89.9 | **23.5** | 46.8 | 90.1 | 20.3 | 22.1 | 90.1 | 7.0 | 37.5 | 90.0 | 15.3 |
| **PPA.T** | Low | 5.5 | 98.0 | 4.7 | **12.4** | 98.0 | **13.1** | 9.7 | 98.0 | 10.2 | 2.6 | 98.1 | 0.9 | 7.7 | 98.0 | 7.5 |
|  | Medium | 13.8 | 94.0 | 6.3 | **23.5** | 94.0 | **13.7** | 19.4 | 94.1 | 10.7 | 9.6 | 94.1 | 3.0 | 16.4 | 94.0 | 8.3 |
|  | High | 21.1 | 90.1 | 7.3 | **30.8** | 90.0 | **13.1** | 25.3 | 90.1 | 9.9 | 15.4 | 90.1 | 3.5 | 22.9 | 89.8 | 8.1 |
| **PPA.Y** | Low | 4.3 | 96.6 | 1.2 | **9.5** | 95.9 | **6.2** | 8.9 | 95.3 | 4.5 | 7.4 | 95.0 | 2.6 | 7.7 | 95.0 | 2.9 |
|  | Medium | 10.7 | 91.3 | 1.7 | **17.9** | 90.9 | **7.1** | 15.1 | 90.9 | 4.9 | 12 | 91.1 | 2.5 | 14.8 | 90.8 | 4.6 |
|  | High | 17.9 | 85 | 2.0 | **28.4** | 85.0 | **8.8** | 23.4 | 85.0 | 5.6 | 20 | 85.0 | 3.3 | 23.8 | 85.0 | 5.8 |

* In the table, the best performance is highlighted in bold, while the second-best and third-best performance is highlighted with underlines.

**Table S4**. **Performance comparison between different predictors of kinase-specific phosphorylation sites, evaluated in terms of Sensitivity (SE), Specificity (SP) and the Matthews correlation coefficient ­(MCC).**

|  |  | **GPS 3.0** | | | **MusiteDeep** | | | **Musite 1.0** | | | **NetPhos 3.1** | | |
| --- | --- | --- | --- | --- | --- | --- | --- | --- | --- | --- | --- | --- | --- |
| **Datasets** | Cutoff threshold | SE | SP | MCC | SE | SP | MCC | SE | SP | MCC | SE | SP | MCC |
| **AGC/PKA** | Low | 46.4 | 98.1 | 33.8 | **86.7** | 98.0 | **57.0** | 64.1 | 98.0 | 44.2 | 50.3 | 98.0 | 35.6 |
|  | Medium | 71.8 | 94.0 | 30.8 | **95.5** | 93.9 | **40.4** | 86.2 | 94.1 | 37.2 | 70.2 | 94.0 | 30.1 |
|  | High | 82.9 | 90.0 | 27.6 | **96.1** | 90.0 | **32.3** | 87.8 | 90.0 | 29.4 | 77.9 | 90.0 | 25.8 |
| **AGC/PKC** | Low | 18.4 | 97.9 | 15.4 | **82.1** | 98.0 | **61.8** | 32.1 | 98.0 | 27.4 | 15.8 | 98.0 | 13.5 |
|  | Medium | 37.2 | 94.0 | 18.3 | **89.3** | 94.0 | **45.4** | 53.6 | 94.0 | 27.3 | 31.6 | 94.0 | 15.2 |
|  | High | 57.7 | 90.0 | 22.3 | **92.9** | 89.9 | **37.4** | 67.3 | 90.1 | 26.7 | 42.9 | 90.0 | 15.6 |
| **CMGC/CDK** | Low | 42.3 | 97.9 | 34.5 | **90.1** | 98.0 | **66.1** | 49.3 | 98.1 | 40.7 | 33.8 | 98.0 | 28.7 |
|  | Medium | 74.6 | 93.8 | 37.6 | **100** | 93.9 | **50.2** | 73.9 | 94.1 | 37.9 | 65.5 | 94.0 | 33.5 |
|  | High | 80.3 | 90.0 | 32.2 | **100** | 89.9 | **40.4** | 76.8 | 90.1 | 30.8 | 76.8 | 90.0 | 30.7 |
| **Other/CK2** | Low | 49.3 | 98.0 | 42.4 | **73.5** | 97.7 | **56.6** | 49.3 | 97.9 | 42.0 | 39.0 | 98.0 | 34.8 |
|  | Medium | 72.8 | 94.0 | 39.9 | **83.8** | 94.0 | **45.6** | 75.0 | 94.1 | 41.2 | 64.0 | 94.0 | 35.1 |
|  | High | 79.4 | 90.0 | 34.2 | **90.4** | 89.4 | **38.2** | 83.1 | 90.1 | 36.1 | 75.0 | 90.1 | 32.3 |
| **TK/SRC** | Low | **28.9** | 96.0 | **29.1** | - | - | - | 27.4 | 96.0 | 27.7 | 20.0 | 96.1 | 20.2 |
|  | Medium | **51.9** | 91.2 | **36.5** | - | - | - | 38.5 | 91.2 | 26.3 | 38.5 | 91.1 | 26.2 |
|  | High | 59.3 | 85.4 | 32.3 | - | - | - | 51.9 | 85.1 | 27.0 | 51.9 | 85.1 | 27.0 |

**Table S4**. **Performance comparison between different predictors of kinase-specific phosphorylation sites, evaluated in terms of Sensitivity (SE), Specificity (SP) and the Matthews correlation coefficient ­(MCC). (Continue)**

|  |  | **KinasePhos 2.0** | | | **PhosphoPredict** | | | **PhosphoPick** | | | **PhosContext2vec** | | |
| --- | --- | --- | --- | --- | --- | --- | --- | --- | --- | --- | --- | --- | --- |
| **Datasets** | Cutoff threshold | SE | SP | MCC | SE | SP | MCC | SE | SP | MCC | SE | SP | MCC |
| **AGC/PKA** | Low | 19.3 | 98.0 | 13.9 | 56.9 | 98.2 | 41.2 | 39.8 | 98.0 | 28.6 | 44.4 | 98.0 | 31.8 |
|  | Medium | 32.0 | 94.0 | 12.6 | 60.8 | 94 | 25.9 | 45.9 | 94.0 | 19.1 | 78.3 | 93.6 | 32.7 |
|  | High | 40.3 | 90.0 | 11.7 | 72.4 | 90.2 | 24 | 47.5 | 90.0 | 14.5 | 85.0 | 90.0 | 28.5 |
| **AGC/PKC** | Low | 16.3 | 97.9 | 13.6 | 19.4 | 98 | 16.6 | 23.5 | 98.0 | 20.3 | 35.4 | 98.0 | 30.1 |
|  | Medium | 22.4 | 94.0 | 9.9 | 21.4 | 97.3 | 15.7 | 35.7 | 94.0 | 17.5 | 73.3 | 94.0 | 37.4 |
|  | High | 27.0 | 90.0 | 8.1 | 29.6 | 90 | 9.4 | 47.4 | 90.0 | 17.6 | 82.1 | 89.3 | 31.9 |
| **CMGC/CDK** | Low | 4.9 | 98.0 | 3.0 | 39.4 | 98.1 | 33.4 | 35.9 | 98.0 | 30.2 | 33.1 | 98.0 | 28.2 |
|  | Medium | 9.2 | 94.0 | 1.9 | 54.2 | 96 | 33.5 | 47.2 | 93.9 | 23.6 | 82.4 | 94.0 | 42.0 |
|  | High | 14.8 | 90.0 | 2.3 | 54.2 | 96 | 33.5 | 50.7 | 90.0 | 19.1 | 91.5 | 89.8 | 36.6 |
| **Other/CK2** | Low | 54.4 | 97.9 | 45.4 | 21.3 | 98 | 19.8 | - | - | - | 36.8 | 98.0 | 32.9 |
|  | Medium | 72.1 | 93.7 | 38.7 | 39.7 | 94.1 | 21.5 | - | - | - | 65.4 | 93.8 | 35.4 |
|  | High | 86.8 | 89.8 | 37.4 | 44.9 | 90.1 | 17.9 | - | - | - | 76.5 | 89.9 | 32.7 |
| **TK/SRC** | Low | 18.5 | 95.7 | 17.4 | 14.8 | 95.9 | 13.7 | 9.6 | 95.1 | 5.9 | 20.7 | 95.9 | 20.4 |
|  | Medium | 25.2 | 91.1 | 15.0 | 23.7 | 91 | 13.6 | 14.8 | 90.9 | 5.5 | 30.4 | 91.0 | 19.3 |
|  | High | 32.6 | 84.6 | 12.8 | 27.4 | 85.3 | 9.7 | 23.7 | 84.4 | 6.2 | **63.7** | 84.8 | **34.5** |

* In the table, the best performance is highlighted in bold, while the second-best performance was highlighted with underlines.

**SUPPLEMENTARY FIGURES**

**Figure S1** shows the performance comparison results in accordance with the selection of different hyper-parameters. The two hyper-parameters are the penalty parameter *C* and the kernel type *K* in SVM models. We evaluated the performance results based on the average AUCs in 10-fold cross-validation.


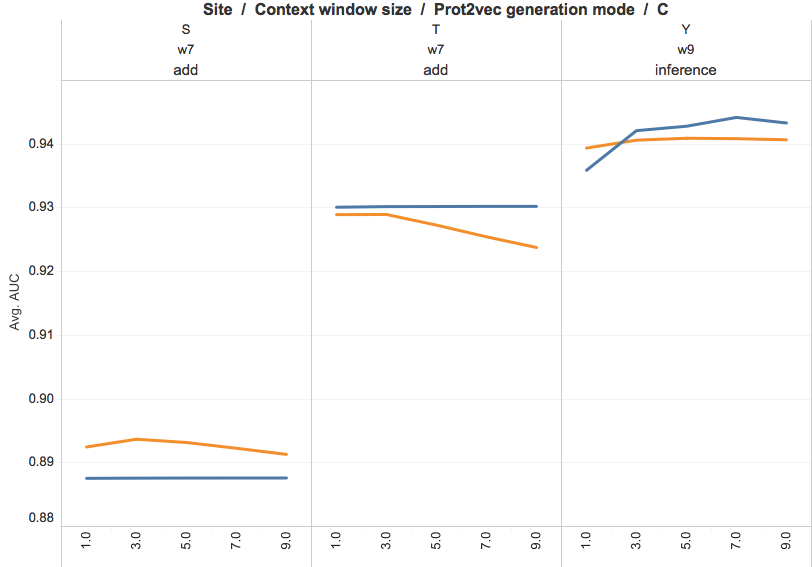


1. Hyper-parameter tuning in general phosphorylation site prediction


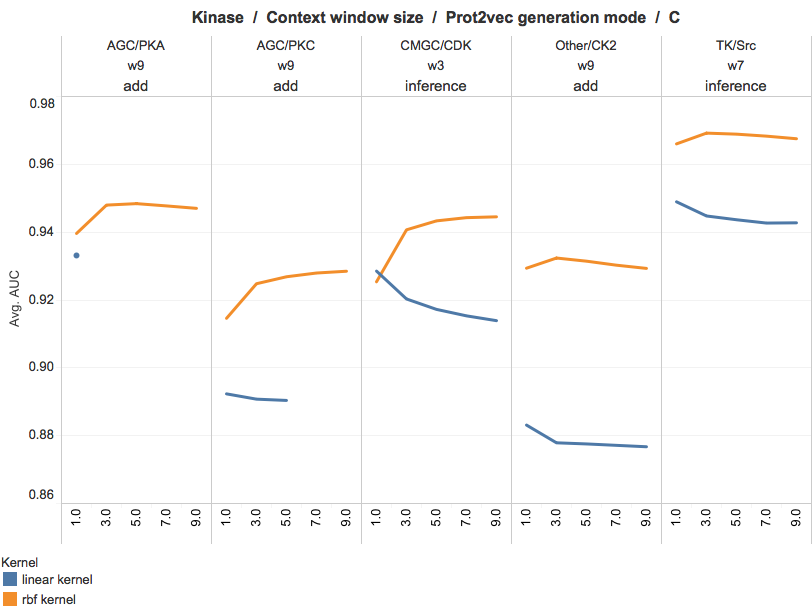


1. Hyper-parameter tuning in kinase-specific phosphorylation site prediction

**Figure S1. Hyper-parameter tuning for SVM models trained with distributed contextual feature vectors.** The contextual window size and the contextual representation were used in accordance with the results demonstrated in **Figure 2** and **Figure 3** in the main manuscript. “add” and “inference” indicate the *context2vec^add^* and *context2vec^inference^* representations, respectively. “w” indicates the contextual window size. Note that the results of models that did not converge during training were not included in this figure.

**Figure S2** depicted the architecture of the PhosContext2vec web server. Due to the lengthy running time of PSI-BLAST, an asynchronous task scheduler was incorporated to release the backend server from long running jobs. In this way, users can safely close the request window and still receive results by providing email addresses.

**
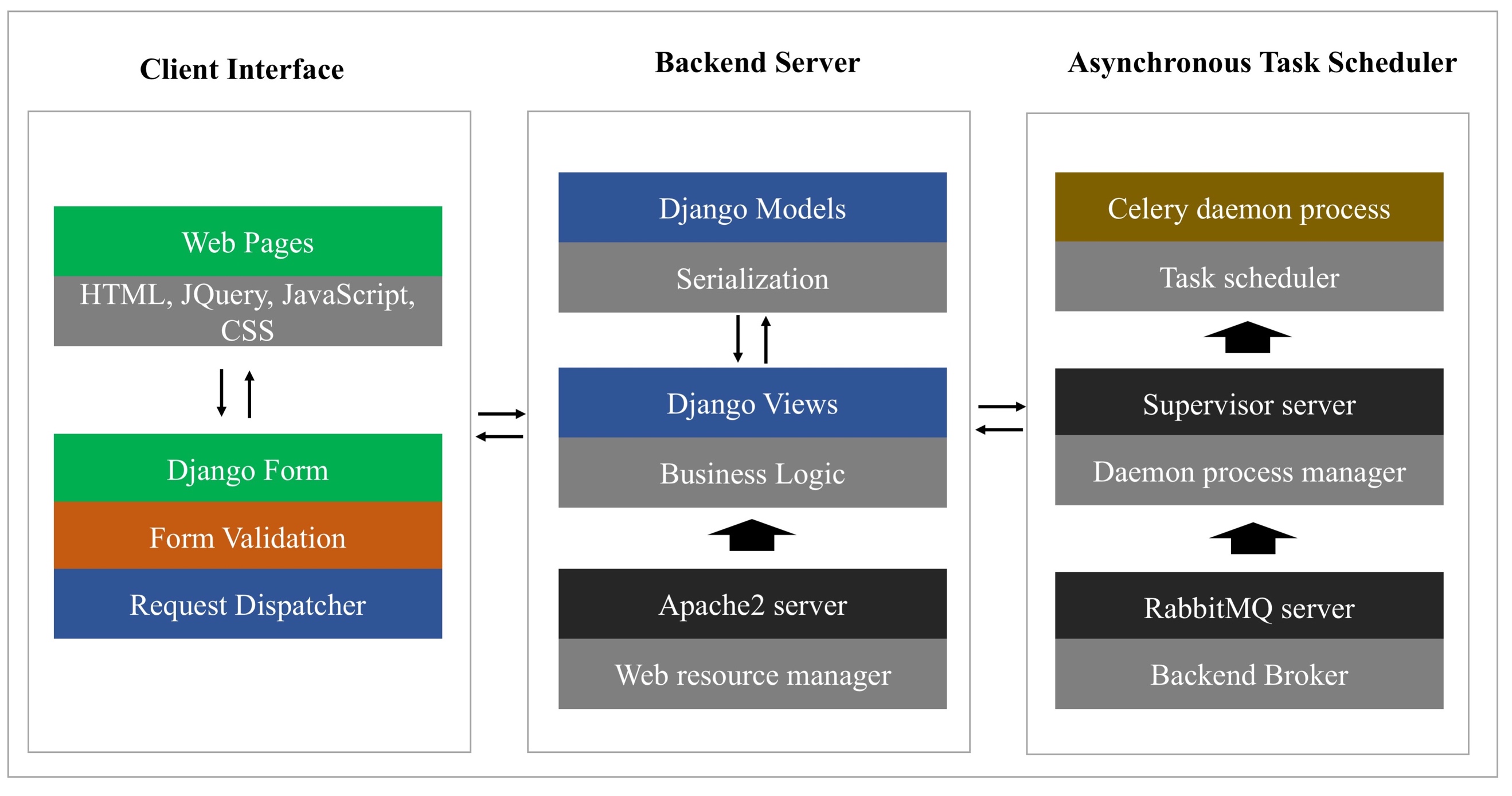
**

**Figure S2. The architecture of the PhosContext2vec web server.** The web server is composed of three modules, where the client interface accepts and forwards the submitted requests to backend server, the backend server determines the logic of each request and invokes actions on corresponding models, and the asynchronous task scheduler manager can interact with the web server resources asynchronously so that long running jobs can be executed simultaneously.

**SUPPLEMENTARY EXAMPLES**

***Task 1. Generating contextual feature vector for protein sequences***


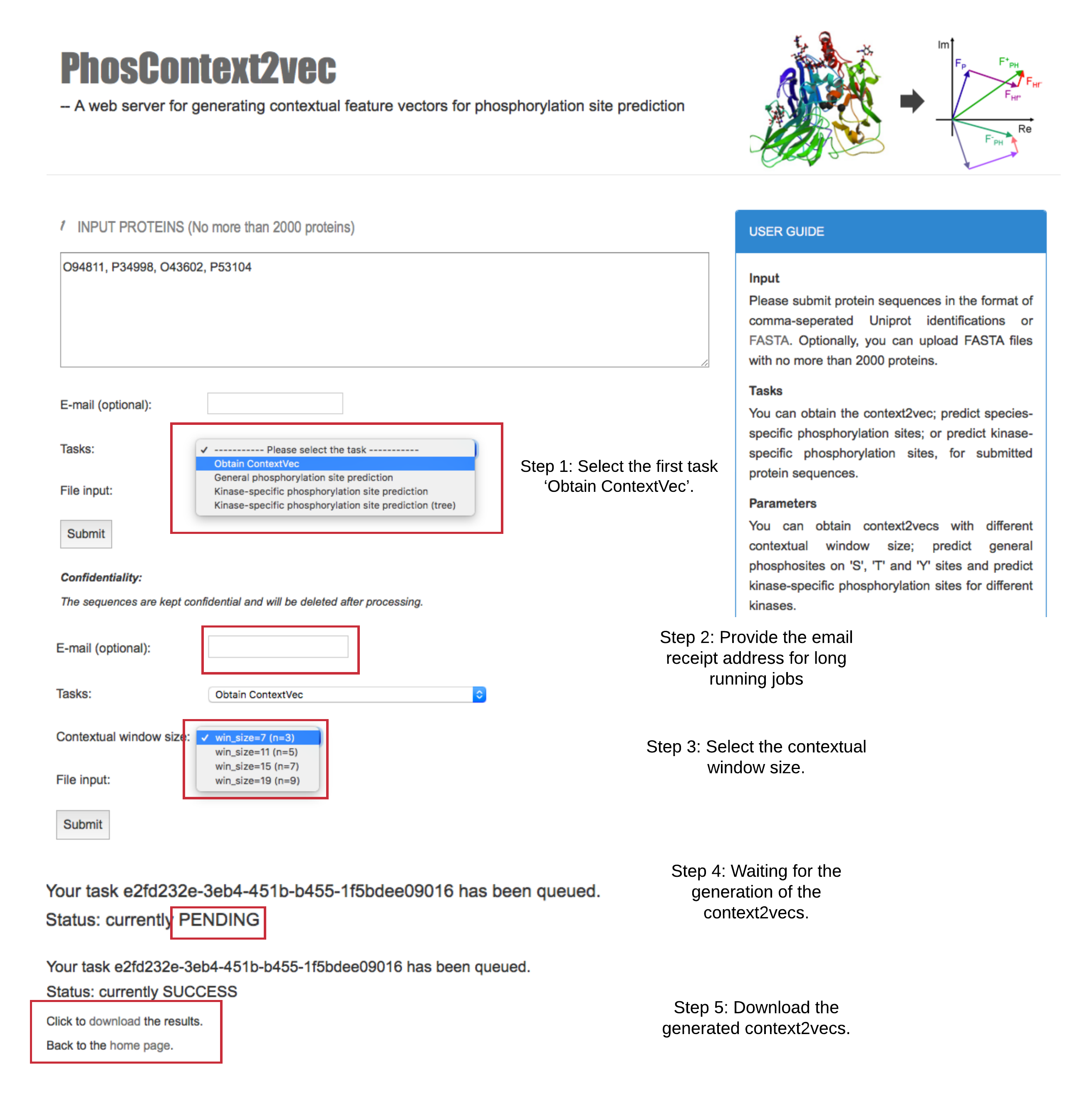


**Figure S3 (a)**. An example of using PhosContext2vec service for generating the contextual vector for the submitted protein sequences (UniProt IDs: O94811, P34998, O43602, and P53104).

***Task 2. Predicting general phosphorylation site for protein sequences***


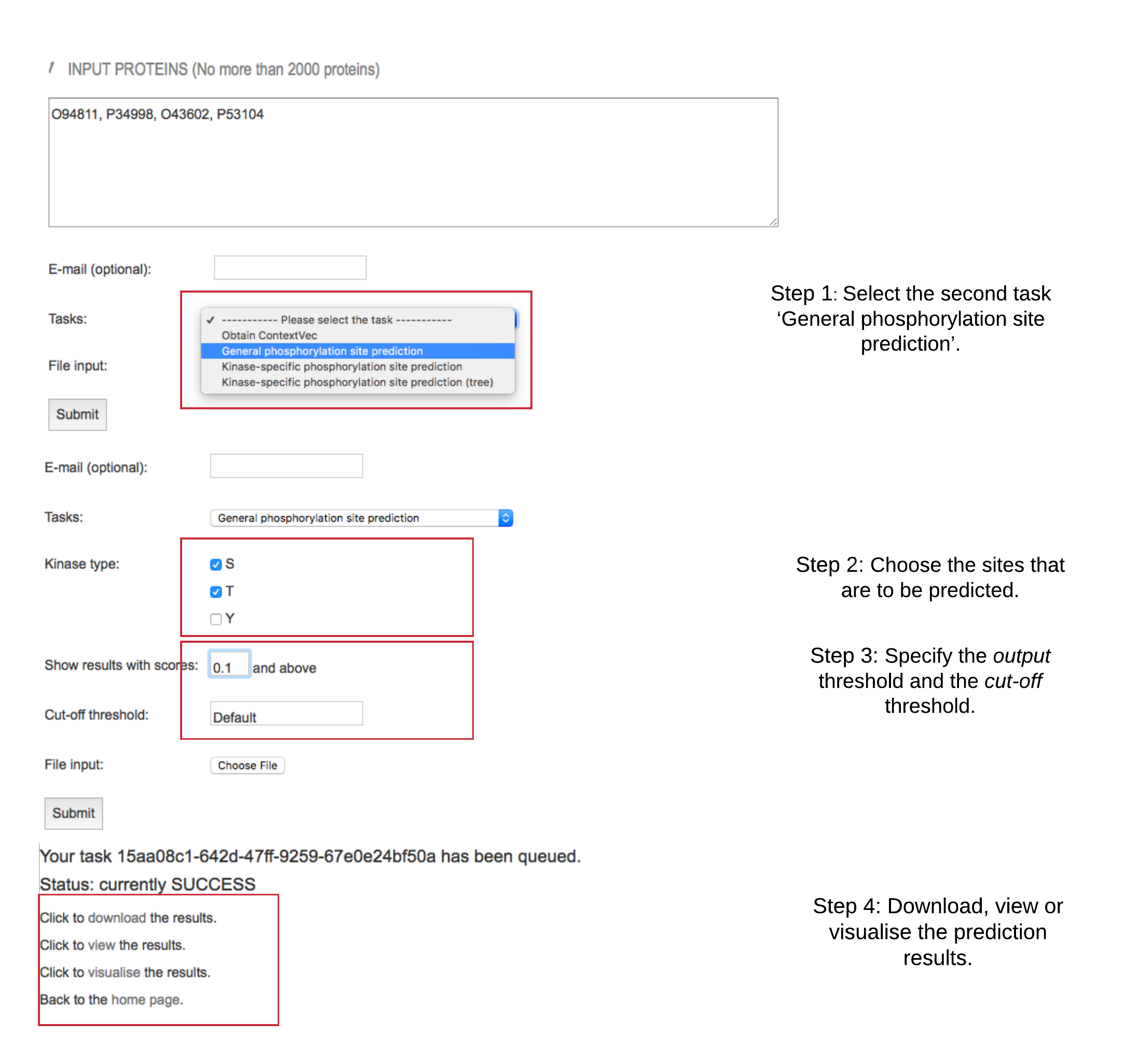


**Figure S3 (b)**. An example of using PhosContext2vec server for predicting general phosphorylation sites for the submitted protein sequences (UniProt IDs: O94811, P34998, O43602 and P53104).

***Task 3. Predicting kinase-specific phosphorylation site prediction***


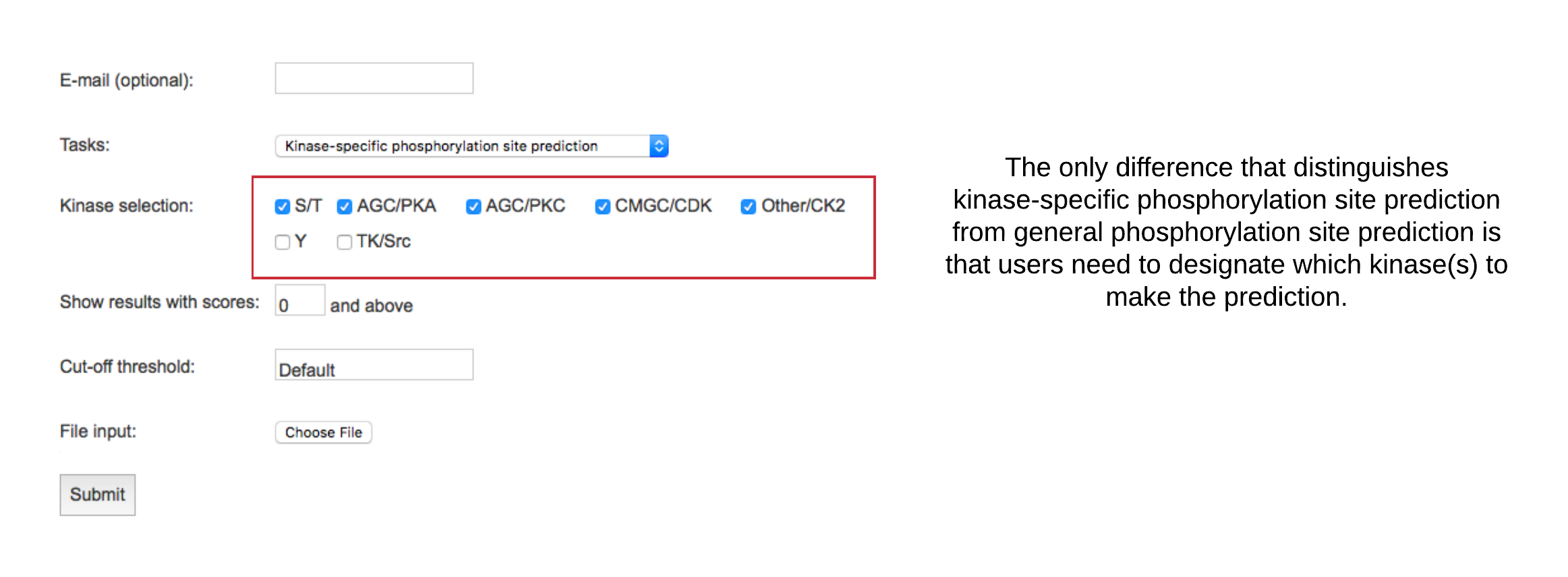


**Figure S3 (c)**. An example of using the web server to predict kinase-specific phosphorylation sites for the submitted protein sequences (UniProt IDs: O94811, P34998, O43602 and P53104).

**Description of the output of the three different tasks**

For the html webpage-based output of phosphorylation site prediction, a summary report is provided in a table for each of the submitted protein sequences. The prediction output consists of the following seven columns:

- Position: the position of the predicted phosphorylation site in the submitted protein sequence;
- Code: the one-letter amino acid residue of the predicted phosphorylation site;
- Kinase: the corresponding kinase type whose potential phosphorylation sites will be predicted;
- Contexts: the contextual subsequence used by the server for generating the prot2vec features;
- Score: the probability score for the predicted phosphorylation site;
- Cut-off: the designated prediction cutoff value;
- Annotation: this column indicates whether a residue is predicted to be a phosphorylation site. “Yes” indicates that a given site is a positive prediction.

For the graph-based output of phosphorylation site prediction, a graphical representation is generated for each submitted sequence. In the graphical output, different colors represent different phosphorylation sites or kinases, the Y-axis represents the probability scores for the given site, while the X-axis represents the residue position of the predicted phosphorylation site in the submitted sequence. **Figure S4 (a)** and **(b)** below provide respective examples of the html-based and the graph-based outputs for the protein sequence (UniProt ID: O43602).


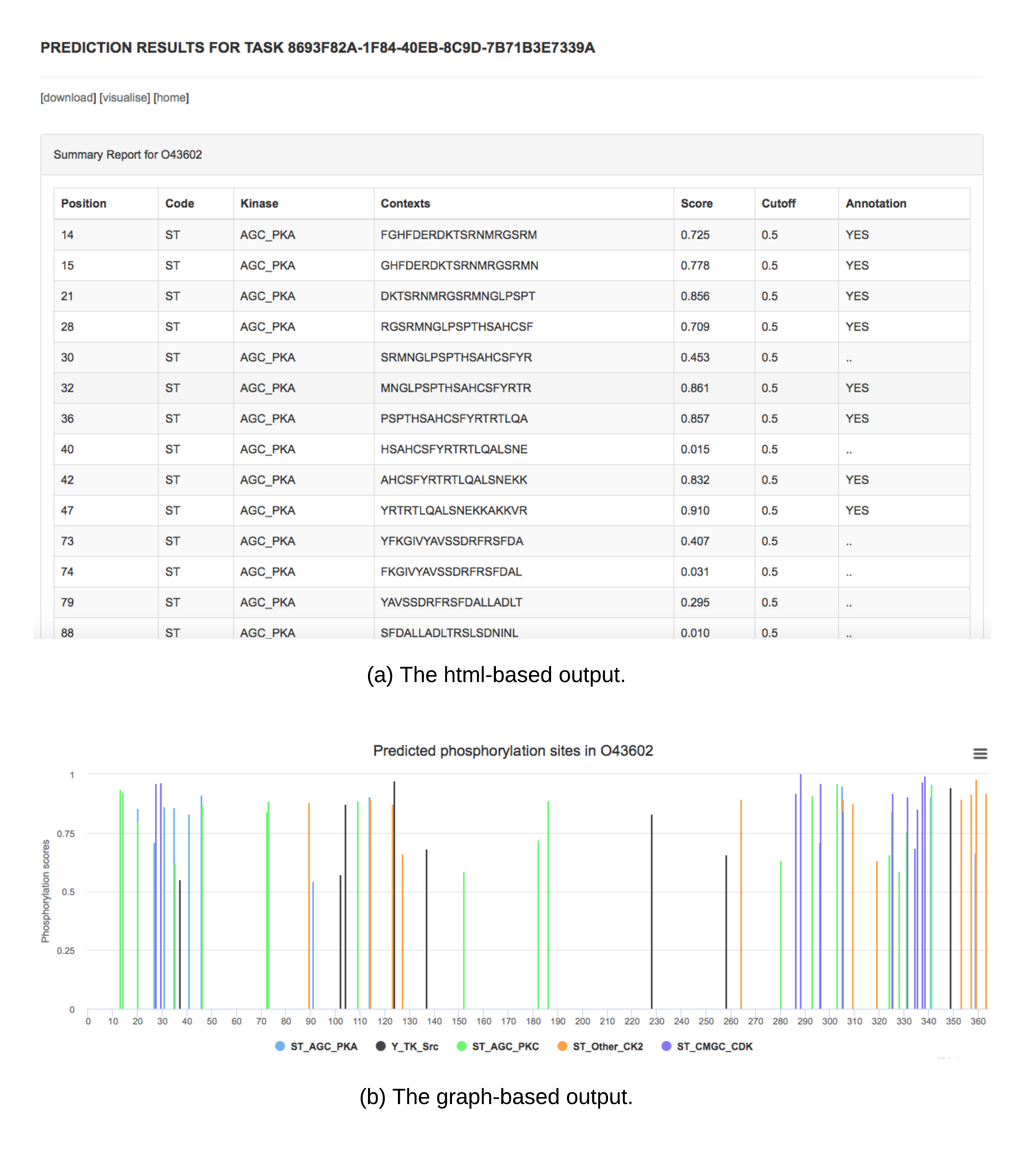


**Figure S4**. **(a)** The html webpage-based and **(b)** the graph-based outputs of kinase-specific phosphorylation site predictions for one submitted protein (UniProt ID: O43602).

**References:**

1. Xue, Y., et al., *GPS 2.0, a tool to predict kinase-specific phosphorylation sites in hierarchy.* Molecular & cellular proteomics, 2008. **7**(9): p. 1598-1608.

2. Consortium., U., *UniProt: a hub for protein information.* Nucleic Acids Research, 2015. **43**(D1): p. D204-D212.

3. Dinkel, H., et al., *Phospho. ELM: a database of phosphorylation sites—update 2011.* Nucleic acids research, 2011. **39**(suppl 1): p. D261-D267.
